# Transcriptomics and proteomics analyses of the PACAP38 influenced ischemic brain in permanent middle cerebral artery occlusion model mice

**DOI:** 10.1186/1742-2094-9-256

**Published:** 2012-11-23

**Authors:** Motohide Hori, Tomoya Nakamachi, Randeep Rakwal, Junko Shibato, Tetsuo Ogawa, Toshihiro Aiuchi, Tatsuaki Tsuruyama, Keiji Tamaki, Seiji Shioda

**Affiliations:** 1 Department of Forensic Medicine and Molecular Pathology, School of Medicine, Kyoto University, Kyoto, 606-8315, Japan; 2 Department of Anatomy I, School of Medicine, Showa University, 1-5-8 Hatanodai, Shinagawa, Tokyo, 142-8555, Japan; 3 Department of Center for Biotechnology, Showa University, 1-5-8 Hatanodai, Shinagawa, Tokyo, 142-8555, Japan; 4 Graduate School of Life and Environmental Sciences, University of Tsukuba, Tsukuba, 305-8572, Japan

**Keywords:** CRMP2, Crtam, DNA microarray, Gabra6, Ischemia, *Il6*, Neuroprotection, PACAP

## Abstract

**Introduction:**

The neuropeptide pituitary adenylate cyclase-activating polypeptide (PACAP) is considered to be a potential therapeutic agent for prevention of cerebral ischemia. Ischemia is a most common cause of death after heart attack and cancer causing major negative social and economic consequences. This study was designed to investigate the effect of PACAP38 injection intracerebroventrically in a mouse model of permanent middle cerebral artery occlusion (PMCAO) along with corresponding SHAM control that used 0.9% saline injection.

**Methods:**

Ischemic and non-ischemic brain tissues were sampled at 6 and 24 hours post-treatment. Following behavioral analyses to confirm whether the ischemia has occurred, we investigated the genome-wide changes in gene and protein expression using DNA microarray chip (4x44K, Agilent) and two-dimensional gel electrophoresis (2-DGE) coupled with matrix assisted laser desorption/ionization-time of flight-mass spectrometry (MALDI-TOF-MS), respectively. Western blotting and immunofluorescent staining were also used to further examine the identified protein factor.

**Results:**

Our results revealed numerous changes in the transcriptome of ischemic hemisphere (ipsilateral) treated with PACAP38 compared to the saline-injected SHAM control hemisphere (contralateral). Previously known (such as the interleukin family) and novel (*Gabra6*, *Crtam*) genes were identified under PACAP influence. In parallel, 2-DGE analysis revealed a highly expressed protein spot in the ischemic hemisphere that was identified as dihydropyrimidinase-related protein 2 (DPYL2). The DPYL2, also known as Crmp2, is a marker for the axonal growth and nerve development. Interestingly, PACAP treatment slightly increased its abundance (by 2-DGE and immunostaining) at 6 h but not at 24 h in the ischemic hemisphere, suggesting PACAP activates neuronal defense mechanism early on.

**Conclusions:**

This study provides a detailed inventory of PACAP influenced gene expressions and protein targets in mice ischemic brain, and suggests new targets for thereaupetic interventions.

## Introduction

In 1989, a new hypothalamic hormone with the significant ability to activate adenylate cyclase in rat pituitary cell cultures was discovered and aptly named pituitary adenylate cyclase-activating polypeptide or PACAP [1,2]. As reported in those first papers, PACAP exists in two amidated forms, PACAP38 and its truncated form, PACAP27. The ovine PACAP38 is 176 amino acids long, proceeded by a putative signal peptide and a proregion (107 amino acids), and followed by a Gly-Arg-Arg sequence for proteolytic processing and C-terminal amidation; the human PACAP38 has been shown to be identical to the isolated ovine PACAP [3]. The sequence of PACAP38 encompasses an internal cleavage-amidation site (Gly28-Lys29-Arg30), suggesting that it can generate a 27 residue-amidated polypeptide fragment or PACAP27. Similarly, the rat (PACAP precursor that is highly similar to the ovine and human PACAP precursors) and the mouse (PACAP precursor shows 81 to 93% sequence similarity) PACAP cDNAs were cloned (reviewed in [4]).

To briefly note some characteristics, PACAP is a member of the secretin, glucagon, and vasoactive intestinal polypeptide (VIP) family showing the most homology to VIP; all peptides are present in the tissue but PACAP38 is the most dominant form [5]. An important finding was on the potency of adenylate cyclase activation by PACAP 1,000 to 10,000 times greater than VIP, as demonstrated in pituitary cell, neuron, and astrocyte cultures. PACAP and its receptors (PAC1, VPAC1, and VPAC2) are widely distributed in the brain (central nervous system, CNS) and peripheral organs (notably endocrine pancreas, gonads, respiratory, and urogenital tracts). Consequently, PACAP exerts pleiotropic effects including control of neurotransmitter release, vasodilation, bronchodilation, activation of intestinal motility, increase in insulin and histamine secretion, immune modulation, and stimulation of cell proliferation and/or differentiation. These and other characteristics and functions of PACAP are comprehensively reviewed by Arimura [4] and Vaudry *et al*. [6].

PACAP is now considered a potent neurotrophic and neuroprotective peptide [7-11]. The specific role of PACAP in neuroprotection is of interest to our study, and forms the basis for the present experiments with an aim to unravel new potential targets of PACAP at the gene and/or protein level, and possible mechanisms involved therein. PACAP exerts potent neuroprotective effects not only *in vitro* but also in *in vivo* models of Parkinson's disease, Huntington's disease, traumatic brain injury, and stroke. The neuroprotective effects of PACAP are based on its capacity to prevent neuronal apoptosis by acting directly on neurons or indirectly through the release of neuroprotective factors by astrocytes [12,13]. These biological activities are mainly mediated through activation of the PAC1 receptor which is currently considered a potential target for the treatment of neurodegenerative diseases. However, the use of native PACAP, the endogenous ligand of PAC1, as an efficient neuroprotective drug is actually limited by its rapid degradation. Moreover, injection of PACAP in humans induces peripheral side effects that are mainly mediated through VPAC1 and VPAC2 receptors [7].

The intraventricular infusion of PACAP38 at 1 pmol/h significantly delayed neuronal cell death in the hippocampal CA1 region that would normally occur a couple of days after induction of ischemia. The intravenous administration of PACAP (only 16 pmol/h) decreased delayed neuronal cell death in the hippocampus. Subsequently, another study showed that PACAP quickly crosses the blood–brain barrier at approximately 0.12% by a saturable system. PACAP prevented delayed neuronal cell death even when it was given intravenously 24 h after ischemia induction. Moreover, anti-apoptotic factors and anti-cell-death pathways were increased by PACAP and decreased by the addition of the PAC1R antagonist, PACAP 6–38, after ischemia and in other *in vitro* studies. The studies suggest that the neuroprotection by PACAP depends on the activation of the PAC1R, and exogenous PACAP might extend the therapeutic time window for treatment of ischemia-related conditions, such as stroke [10].

In an effort to unravel PACAP molecular targets in the brain, especially under ischemia, we further advanced our research using the established permanent middle cerebral artery occlusion (hereafter referred to as PMCAO) model mice and the optimized DNA microarray approach with the 44K mouse whole genome chip [14]. Our study employed the intraluminal filament technique PMCAO model because of its simplicity and noninvasive characteristics compared to the classical electrocoagulation method for PMCAO. The transient MCAO results in reperfusion injury, and to avoid this complication, the method of choice was in favor of intraluminal PMCAO. Further, we also applied the proteomics approach [15,16] to examine the protein changes in the same sample/brain hemisphere as was used for the transcriptomics study.

We used DNA microarray analysis to answer two questions. 1) What are the PACAP influenced genes? 2) Can we pinpoint the transcripts specifically altered (increased or decreased) by PACAP, that is, the genes that are not the result of ischemia itself, and are predominantly regulated by PACAP? By proteomics, we aimed to identify proteins from the two-dimensional gel profile that was differentially regulated by PACAP under the ischemic condition. Our results reveal numerous potential gene candidates, such as interleukin family members, *Gabra6*, and *Crtam* that might be involved in PACAP-mediated neuroprotective mechanisms. The identification of an important protein involved in neuronal function, the collapsin response mediator protein (CRMP2) [17], was another key finding of a PACAP-regulated protein in the ischemic hemisphere. Results presented show the usefulness of omics approaches in screening of potential targets of PACAP-regulated genes and proteins.

## Methods

### Animals and husbandry

Animal care and experimental procedures were used as approved by the Institutional Animal Care and Use Committee of Showa University (School of Medicine), Tokyo, Japan. Thirty male mice (C57BL/6J), 9-weeks-old, body weight 25 to 35 g, were purchased from Charles River (Kanagawa, Japan). Mice were housed at the Animal Institution in Showa University in acrylic cages (eight mice/cage) maintained at 23°C with a standard 12-h light/dark cycle, optimum humidity, and temperature control. Animals were given access to tap water and laboratory chow *ad libitum*.

### Permanent middle cerebral artery occlusion (PMCAO), PACAP treatment, and SHAM control

**Figure 1:**
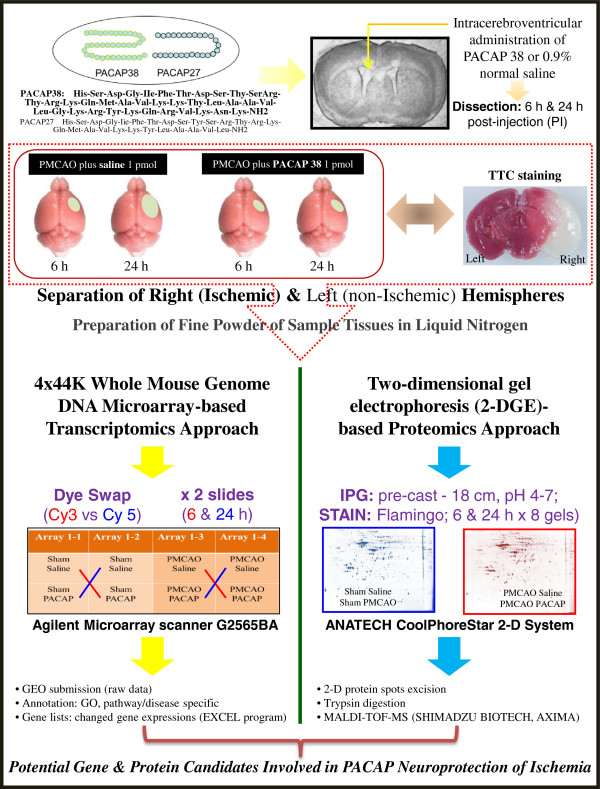
**The experimental outline and workflow.** Effect of intracerebroventricular administration of pituitary adenylate cyclase-activating polypeptide (PACAP)-38 into the ischemic mouse brain (permanent middle cerebral artery occlusion, PMCAO model) is evaluated at the molecular level in the ipsilateral (right) hemisphere. Sham control treated with saline is used for the comparison. TTC staining shows he ischemic region in the brain. The ipsilateral hemisphere is sampled and finely powdered in liquid nitrogen, followed by investigation into molecular level changes at the level of gene and protein expressions by DNA microarray and proteomics approaches, respectively.

The experimental design is presented in Figure 1. The PMCAO-model mice were generated as described previously [14]. Briefly, the mice were anesthetized with 4% sevoflurane (induction) and 2% sevoflurane (maintenance) in a 30% O_2_ and 70% N_2_O gas mixture via a face mask. An incision was then made in the cervical skin followed by opening of the salivary gland, and the right common carotid artery was visualized. A midline cervical incision was made to expose the external carotid artery. The intraluminal filament technique was used, as reported by Hori *et al*. [14] to generate the PMCAO model. The PACAP38 (1 μL containing 1 pmol) or 1 μL of saline (0.9% NaCl) was injected intracerebroventrically, immediately after PMCAO. PACAP38 (Peptide Institute Inc., Osaka, Japan; supplier temperature was −20°C) was dissolved at 10^-5^M concentration by saline, and stored at −80°C. PACAP test solution (for injection) was diluted × 10 times with 0.9% NaCl just before use. In the sham control animals, the wound was sutured following exposure of the external carotid artery. The PACAP38 or saline was injected intracerebroventrically in the same concentrations as above. After injection, the animals were returned to their cages. A total of eight groups were prepared: four groups of three, seven, four and five mice in the PMCAO plus PACAP38 and PMCAO plus saline cohorts at 6 and 24 h after the operation, respectively, and five mice each in the control (sham) plus PACAP and saline groups at 6 and 24 h after operation, respectively (see Additional file 1: Table S1). We used three mice each in PMCAO groups that exhibited neurological grades G1 and G2 [13,14], and three mice each at random in sham groups for the subsequent downstream analysis. Some of the mice were examined for ischemia by TTC (2, 3, 5-triphenyltetrazolium chloride) staining of brain sections (2-mm slices) at 37°C for 10 minutes [13,14,18].

### Dissection of brain, sampling and storage, extraction of total RNA, cDNA synthesis, and semiquantitative RT-PCR

Six or 24 hours post-injection of PACAP38 or saline, the mice were removed from their cages, decapitated, and their brains carefully removed on ice. The left (contralateral) and right (ipsilateral; ischemic) hemispheres were dissected and placed in 2-mL Eppendorf tubes, which were then quickly immersed in liquid nitrogen before being stored at −80°C prior to further analysis (Figure 1). Stored tissues were ground to a very fine powder with liquid nitrogen, and used for total RNA extraction, followed by a quantity and quality check [14-16]. To validate the total RNA quality and subsequently synthesized cDNA, RT-PCR was carried out using two commonly used housekeeping genes *glyceraldehyde 3-phosphate dehydrogenase* (*GAPDH*) and *beta-actin* as positive controls [14,19]. The 3’-UTR gene-specific primers were designed (see Additional file 2: Table S2). The cDNA synthesis and RT-PCR analysis protocol used is as follows: Total RNA samples were first DNase-treated with RNase-free DNase (Stratagene, Agilent Technologies, La Jolla, CA, USA). First-strand cDNA was then synthesized in a 20 μL reaction mixture with an AffinityScript QPCR cDNA Synthesis Kit (Stratagene) according to the protocol provided by the manufacturer, using 1 μg total RNA. The reaction conditions were: 25°C for 5 minutes, 42°C for 5 minutes, 55°C for 40 minutes and 95°C for 5 minutes. The synthesized cDNA was made up to a volume of 50 μL with sterile water supplied in the kit. The reaction mixture contained 0.6 μL of the first-strand cDNA, 7 pmol of each primer set and 6.0 μL of the Emerald Amp PCR Master Mix (2X premix) (TaKaRa Shuzo, Shiga, Japan) in a total volume of 12 μL. Thermal-cycling (Applied Biosystems, Tokyo, Japan) parameters were as follows: after an initial denaturation at 97°C for 5 minutes, samples were subjected to a cycling regime of 20 to 40 cycles at 95°C for 45 s, 55°C for 45 s, and 72°C for 1 minute. At the end of the final cycle, an additional extension step was carried out for 10 minutes at 72°C. After completion of the PCR the total reaction mixture was spun down and mixed (3 μL), before being loaded into the wells of a 1.2/1.8% agarose fine powder (catalogue number 02468–95, Nacalai Tesque, Kyoto, Japan) gel. Electrophoresis was then performed for approximately 22 minutes at 100 Volts in 1X TAE buffer using a Mupid-ex electrophoresis system (ADVANCE, Tokyo, Japan). The gels were stained (8 μL of 10 mg/mL ethidium bromide in 200 mL 1X TAE buffer) for approximately 7 minutes and the stained bands were visualized with the ChemiDoc XRS+ imaging system (Bio-Rad, 6000 Alfred Nobel Drive, Hercules, CA 94547, USA).

### DNA microarray analysis in the ipsilateral (right) hemisphere

A mouse 4 × 44K whole genome oligo DNA microarray chip (G4122F, Agilent Technologies, Palo Alto, CA, USA) was used for global gene expression analysis using the ipsilateral (ischemic) hemisphere. Briefly, total RNA (900 ng; 300 ng for each replicate pooled) was labeled with either Cy3 or Cy5 dye using an Agilent Low RNA Input Fluorescent Linear Amplification Kit (Agilent). Fluorescent-labeled targets of control (sham) as well as treated (PMCAO) samples with PACAP38 or without PACAP38 (saline) were hybridized to the same microarray slide with 60-mer probes. As illustrated in Figure 1, in this experiment we compared the PMCAO plus PACAP38-injected mice to PMCAO plus saline, that is, the ipsilateral brain region of the PMCAO mice was compared with the same right hemisphere of the control mice. Similarly, sham control plus PACAP38-injected mice to sham control plus saline were analyzed to identify only the PACAP38-influenced genes. A flip labeling (dye-swap or reverse labeling with Cy3 and Cy5 dyes) procedure was followed to nullify the dye bias associated with unequal incorporation of the two Cy dyes into cDNA [14,20]; and references therein].

Briefly, the same total RNA (900 ng) samples were labeled twice with Cy3 or Cy5: a Cy5-labeled treatment (T^Cy5^) and a Cy3-labeled control (C^Cy3^) was hybridized on a slide and then a Cy3-labeled treatment (T^Cy3^) and a Cy5-labeled control (C^Cy5^) were reversely hybridized on another to revise the dye bias associated with unequal incorporation of two Cy dyes into cRNA. Hybridization and wash processes were performed according to the manufacturer’s instructions, and hybridized microarrays were scanned using an Agilent Microarray scanner G2565BA. For the detection of significantly differentially expressed genes between control and treated samples, each slide image was processed by Agilent Feature Extraction software (version 9.5.3.1). Signal ratios of each spot on all slides were normalized by the software and finally, the log ratio of each spot for technical replicates was averaged to give a representative value for each pooled sample and to adjust remaining gene-specific dye bias. The output of microarray analysis used in this study are available under the series number GSE 37565, at the NCBI Gene Expression Omnibus (GEO) public functional genomics data repository (http://www.ncbi.nlm.nih.gov/geo/info/linking.html). To validate the microarray data (ipsilateral hemisphere) RT-PCR, as described above, was also performed on randomly up- and down-regulated genes using 3’-UTR specific gene primers (see Additional file 2: Table S2).

### Extraction of total soluble protein

Total protein was extracted from sample powders (around 50 mg) of the contralateral and ipsilateral hemispheres (control and treatment) using a previously used lysis buffer containing thiourea and Tris (LB-TT) for extraction of brain proteins [15,16]. The composition of slightly modified LB-TT was as follows: 7 M (w/v) urea, 42 g; 2 M (w/v) thiourea, 15.2 g; 4% (w/v) CHAPS, 4.0 g; 18 mM (w/v) Tris–HCl (pH 8.0), 1.8 mL; 14 mM (w/v) Trizma base, 169.5 mg; 0.2% (v/v) Triton X-100, 0.2 mL; 50 mM (w/v) DTT, 771.5 mg; 1% (v/v) pH 3–10 Ampholyte, 1 mL; and two EDTA-free proteinase inhibitor (Roche Diagnostics GmbH, Mannheim, Germany) tablets in a total volume of 100 mL; see Additional file 3: Figure S1 for preparation of LB-TT at room temperature (RT). To extract protein, 1 mL of LB-TT was quickly added to the 2-mL microfuge tube containing the sample powder (immediately after removal from the −80°C deep freezer) and immediately mixed by vortexing (at full speed using a Lab mixer, Iwaki, Tokyo, Japan) for 1 minute at RT. The protein solution in LB-TT was incubated at RT for 30 minutes with mixing by vortexing (for 30 sec) and sonication (for 30 sec in a water bath-type sonicator) for a total of five times. The insoluble protein pellet and/or debris were pelleted by centrifugation at 18,500 g for 15 minutes at 20°C in a high-speed refrigerated micro centrifuge (MX-150, TOMY, Tokyo, Japan). The clear supernatant (around 900 μL) was transferred to a new 1.5-mL microfuge tube, and stored at −80°C as the total soluble protein. Prior to storage, a 0.2 mL aliquot of total protein solution was transferred to a new 2-mL microfuge tube and processed for a second purification, clean up, and concentration step, using the ProteoExtract Protein Precipitation Kit (Calbiochem, Darmstadt, Germany; catalague number 539180). This additional step (see Additional file 4: Figure S2) removes any impurities while concentrating the protein. The pelleted protein, visible as a white solid precipitate at the bottom of the tube, was re-suspended in LB-TT (around 0.2 mL) as above. Protein concentration was determined with a Coomassie Plus^TM^ Protein Assay Kit (PIERCE, Rockford, IL, USA) using bovine serum albumin (BSA) as a standard and a NanoDrop 2000 spectrophotometer (Thermo Scientific, Wilmington, DE, USA).

### Two-dimensional gel electrophoresis and visualization of the separated proteins

The first-dimension separation was carried out using pre-cast IPG strip (pH 4 to 7, 18 cm; GE Healthcare Bio-Sciences AB, Uppsala, Sweden) gels on a CoolPhoreStar IPG-IEF Type-PX unit (Anatech, Tokyo, Japan) followed by the second dimension using hand-cast polyacrylamide gels on an Anatech CoolPhoreStar SDS-PAGE Dual-200 K unit (Anatech, Tokyo, Japan). Two-dimensional gel electrophoresis was performed as essentially described by Toda and Kimura [21]. The detailed protocol for the first- and second dimension-run is described in Additional file 5: Figure S3. After two-dimensional gel electrophoresis, the separated proteins were visualized by staining with Flamingo fluorescent gel stain (catalogue number 161–0491, Bio-Rad). Briefly, the gel was fixed in 200 mL (two-dimensional gel) of fixing solution (40% methanol and 10% acetic acid in MQ water) for 30 minutes at RT, followed by staining with 1X diluted (in MQ water) Flamingo stain (stock of 10X) for 1 h in the dark. The stained proteins were visualized after washing three times for 1 minute each in MQ water with a FluoroPhoreStar 3000 image analysis system (Anatech). The gel images were saved as 8-bit TIFF files for further analysis.

### Protein identification by matrix-assisted laser desorption/ionization-time of flight-mass spectrometry (MALDI-TOF-MS)

Protein spot was excised from the gel using a gel picker (1.8 mm diameter) in combination with a robotic spot analyzer (FluoroPhoreStar 3000, Anatech) and placed in a sterile 0.5 mL microfuge tube (Safe-lock tubes, Eppendorf AG, Hamburg, Germany). Excised protein spots can be stored at 4°C till further analysis. For enzymatic digestion prior to mass spectrometry (MS) analysis 100 μL of 50 mM ammonium bicarbonate (NH_4_HCO_3_) (w/v) solution in 50% acetonitrile (ACN) (v/v) was added to the excised spot in the tube, and rinsed for 10 minutes under constant shaking in a tube shaker at RT. Following spin-down of the washed gel piece, the NH_4_HCO_3_-ACN solution was removed and the gel was vacuum-dried for 30 minutes (Centrifugal vacuum/SpeedVAc evaporator, Eyela, Tokyo, Japan) at RT. To the dried gel pieces, 2 μl of trypsin solution (10 mM NH_4_HCO_3_ and 10 ng/μL of trypsin) was added, and the microfuge tube was kept on ice for 30 minutes, followed by incubation at 37°C for 4 h to overnight for digestion of the protein in gel. Tryptic peptides were extracted with 10 μL of 75% ACN containing 0.025% trifluoroacetic acid (TFA) (v/v) for 15 minutes by ultrasonication (water bath-type). Following a spin-down of any gel debris, the supernatant was analyzed by MS. The supernatant can also be stored at −40°C/-80°C for long-term (1 year) storage.

For MALDI-TOF-MS analysis, we first prepared the matrix solution by dissolving α-Cyano-4-hydroxycinnamic acid (4-CHCA, Shimadzu Biotech, Kyoto, Japan) in 1:1 100% ACN and 0.1% TFA (5 mg of 4-CHCA is dissolved in 500 μL of ACN-TFA solution to give 10 μg/μL 4-CHCA. We next loaded 0.5 μL of the above matrix mixture onto any numbered spot area on the stainless steel MALDI sample plate and left to dry for 5 minutes at RT followed by addition of 1 μL of the peptide sample solution to the same spot, dried for a further 10 to 15 minutes. The ready sample plate was inserted into the vacuum slot of the AXIMA Performance MALDI-TOF-MS (Shimadzu Biotech, Kyoto, Japan) and peptides were identified by Peptide Mass Fingerprint (PMF) analysis. The parent ion masses were measured in the reflectron mode with an accelerating voltage of 24.4 kV. A two-point internal standard for calibration was used with an angiotensin 2 (human, A9525, Sigma; m/z 1046.54 Da) and ACTH fragment 18–39 (human, A0673, Sigma; *m/z* 2465.20 Da). Peptides were selected in the mass range of 900 to 5,000 Da. For data processing, we used MASCOT program (http://www.matrixscience.com). Searching conditions/parameters were fixed (Carbamidomethyl, C) and variable modifications (methionine, M), mass values (monoisotopic), peptide mass (unrestricted), peptide mass tolerance (± 0.3 Da), peptide charge state (1+), and maximum mixed cleavage (1). NCBInr [20120113 (16852130 sequences; 5788275850 residues) (Taxonomy, *Mus musculus* (house mouse) 144612 sequences)] and SWISSPROT [2012_12 (533657 sequences; 189261966 residues) (Taxonomy, *Mus musculus* (house mouse) 16415 sequences)] databases were used.

### Western blot analysis

The separated proteins after SDS-PAGE (mini-gel; Additional file 6: Figure S4) were transferred onto a polyvinyldifluoride (PVDF) (Trans-Blot Turbo Midi PVDF, 0.2 μM, Transfer Packs kit; catalogue number 170–4157). The Trans-Blot Turbo Transfer System (Bio-Rad) was used for the electrotransfer (StandardSD protocol; 25V, 1.0 A, 30 minutes). Following the transfer of proteins on the PVDF membrane (and also confirmed by visualizing all the 10 colored molecular mass standards), it was incubated in 25 mL of 5% blocking solution (Block-Ace powder, catalogue number UK-B80, DS Pharma, Osaka, Japan; Yukizurishi, Sapporo, Hokkaido, Japan) for 1 h under constant slow shaking at RT. Blocking solution was prepared by dissolving 4 g powder in 80 mL 1X TTBS [10X TTBS: NaCl, 80 g; 1M Tris–HCl, pH 7.5, 200 mL; Tween-20, 5 mL]. Western blotting and detection was carried out using the Immun-Star WesternC Chemiluminescent Kit (catalogue number 170–5070, Bio-Rad) following the manufacturer’s instructions. Blocking solution was decanted and the membrane was washed once in 1X TTBS (5 minutes), followed by incubation in 25 mL of primary antibody solution (PAS) (1 μL rabbit anti-CRMP2 protein antibody; catalogue number ab62661; 100 μg, 2 mg/mL; Abcam, http://www.abcam.co.jp) for 1 h, as above. The membrane was then washed with 25 mL of 1X TTBS for five times. After decanting the last TTBS wash, the membrane was incubated in 25 mL of secondary antibody solution (SAS; 0.5 μL of Amersham, ECL anti-rabbit IgG, HRP linked species-specific whole antibody (from Donkey); catalogue number NA 934; GE Healthcare) for 1 h, with slow shaking at RT. The 1X TTBS wash step was repeated five times. For blot development, the luminol/enhancer and peroxide buffer solutions were mixed in a 1:1 ratio (1 mL:1 mL; one membrane volume) and spread over the membrane and incubated at RT for 5 minutes. Excess solution was drained by touching one end of the membrane on a KimWipe paper towel, and the signal (cross-reacting protein bands) was visualized by x-ray film (Kodak, Tokyo, Japan) and on a ChemiDoc XRS+ imaging system. The western blot analysis was repeated at least three times, and representative data from the image obtained using an X-ray film is shown.

### Immunofluorescent staining

The brains were removed and immersed for 1 day in 0.1 M phosphate buffer (PB, pH 7.2) containing 2% paraformaldehyde and then for 2 days in 0.1 M PB containing 20% sucrose, at −80°C (see Additional file 7: Figure S5) [22,23]. The brains were then embedded in the mixture of 20% sucrose in 0.1 M PB and Tissue Tek Optical Cutting Temperature (OCT) solution (2:1; Miles Inc., Elkhart, IN, USA), frozen on dry ice, and stored at −80°C until required. Sections, 8 μm thick, were subsequently cut with a cryostat (HYRAX C50, Microedge Instruments, Tokyo, Japan), and mounted onto gelatin-coated glass slides. Immunofluorescent staining was carried out as described previously [23,24]. Prior to detection of the desired protein, the 8-μm frozen sections were immersed in 0.01 M PBS solution and washed for three times 5 minutes each wash, followed by blocking in 5% normal horse serum (in 0.01 M PBS). Sections were incubated overnight at 4°C in mixtures of primary antibodies, consisting of rabbit anti-CRMP2 antibody (1:2000) with mouse anti-NeuN antibody (1:400) for neuronal cell marker. Post-incubation, the sections were washed with 0.01 M PBS as above. Immunoreactivity for CRMP2 and NeuN was detected using an Alexa 488-labeled goat anti-mouse IgG and an Alexa 546-labeled goat anti-rabbit following 90-minute incubation at RT. After washing with 0.1 M PBS as above, the sections were incubated for 5 minutes with 4′, 6-diamidine-2-phenylindole dihydrochloride (DAPI), 1:10,000 (in 200 mL 0.01 M PBS, add 20 μL DAPI) (Roche Diagnostics, IN, USA) as a nuclear stain. Labeling was imaged with a fluorescence microscope (Axio Imager Z1 with Apotome, Zeiss, Germany). The CRMP2-positive cells were examined in three different regions: healthy, penumbra, and ischemic core in a coronal section of the ipsilateral hemisphere (see Additional file 8: Figure S6). To note, edema (swelling caused by a collection of fluid in the third space surrounding the tissue or organ) was discernible, mostly at the 24 h time period, and is therefore marked in the figure. To assess the specificity of the antibodies, negative control sections in which the primary antibody is absent were prepared to check for background staining levels. Immunostaining were performed on sections obtained from the brains of three mice in each group.

## Results and discussion

### Selection of PMCAO mice with neurological grades between 1 and 2 and preparation of brain tissues for grinding in liquid nitrogen

Mice exhibiting neurological deficiency 6 and 24 hours after PMCAO were graded according to a routinely used methodology in our laboratory [13,18]. Sixteen mice that exhibited neurological grade (NG) 1 and 2 were used for further experiments; two mice died, and one had NG 0 (no PMCAO) and one had NG 3 (extreme neurological symptoms) (Additional file 1: Table S1). The selected mice were decapitated and the brains were dissected on ice. The ipsilateral (right hemisphere, non-injected) and contralateral (left hemisphere; injected with saline or PACAP38) hemispheres without the olfactory bulb (OB) and cerebellum were quickly separated, placed in 2-mL Eppendorf tubes, and deep frozen in liquid nitrogen followed by storage at −80°C. Brains were ground to a very fine powder in liquid nitrogen; aliquots of the finely powdered tissue samples were stored at −80°C, and used for extraction of total RNA or protein thus giving a comparative data expression analysis in the same sample.

### Overview of the brain genomic response to PACAP38 injection in the ischemia brain (ipsilateral hemisphere) by DNA microarray analysis

#### DNA microarray analysis and confirmatory RT-PCR

**Figure 2:**
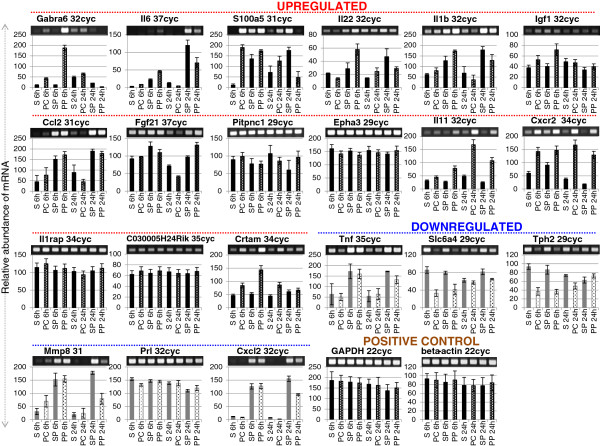
**The mRNA expression profiles of differentially expressed genes.** Both the upregulated and downregulated genes were selected randomly. Gel images on top show the polymerase chain reaction (PCR) product bands stained with ethidium bromide; the band intensities are also presented graphically below for clarity. Lane numbers 1 to 8 indicate sham control (lanes 1, 3, 5, and 7) and permanent middle cerebral artery occlusion (PMCAO) treatment (lanes 2, 4, 6, and 8), respectively. P indicates pituitary adenylate cyclase-activating polypeptide (PACAP) treatment; C is the control (minus PACAP). *GAPDH* and *beta-actin* genes were used a positive control. Semi-quantitative RT-PCR was performed as described in Methods, and the specific 3’-UTR primers are detailed in Additional file 2: Table S2.

To investigate global changes in gene expression in the ischemic hemisphere, the previously optimized protocols for total RNA extraction, critical for any downstream analysis, and DNA microarray analysis in mice were followed (Ogawa *et al*., 2011; [14,20]). The ischemic hemisphere (ipsilateral) consists of the infarct core, penumbra, and non-ischemic region (Additional file 8: Figure S6). Thus, the present experimental design used to perform the microarray analysis provides an overall picture of the ischemic hemisphere rather than one specific ischemic region. Nevertheless, and as will be discussed below, an additional experiment was carried out to check for the expression of proteins in each of the aforementioned regions in the ipsilateral hemisphere. Prior to DNA microarray analysis, the expression of *GAPDH* and *β-actin*, as positive controls, was confirmed at both hemispheres at 6 and 24 h in the PMCAO samples, with or without PACAP; saline was used as control. Results revealed the mRNAs for *GAPDH* and β-actin were expressed almost uniformly across the tested conditions (Figure 2).

DNA microarray analysis gave a wide change in gene expressions as anticipated, and for confirmation of alterations in gene expression by DNA microarray, we randomly selected 14 upregulated genes and 6 downregulated genes with annotated functions; one gene that was the most highly upregulated at 6 h post-ischemia, but with no known function, was also selected. Using RT-PCR, the mRNA expression profiles are presented in Figure 2, which validate the microarray experiment. Two trends can immediately be observed in the upregulated gene candidates examined, namely early (6 h) and late (24 h) expression of target genes by PACAP38 treatment. For example, the gamma-aminobutyric acid (GABA) A receptor, subunit alpha 6, *Gabra6*, was found to be strongly upregulated at 6 h in the PACAP-treated ischemic hemisphere but was not expressed at 24 h. This was confirmed by RT-PCR (Figure 2). This is the first report of *Gabra6* regulation by PACAP. Why PACAP affects *Gabra6* gene expression is unclear, but it may be related to GABA and glutamate levels, which, though not been investigated in this study, are of future interest for investigation. A gene with unknown function, *C030005H24Rik*, showed the most increased mRNA expression fold at 6h, and we analyzed its expression by design-specific primers based on the available gene sequence in the NCBI database. RT-PCR results revealed only a slight increase in mRNA abundance at 6 h (Figure 2). However, as this gene still remains unannotated and there are no functional domains identified so far, the meaning of this gene expression under ischemia remains a mystery. Interestingly, a gene neurofibromatosis 1 (*Nf1*), which is known to regulate PACAP-mediated signaling in astrocytes [25] was found to be slightly induced at 24 h. Therefore, it would be tempting to speculate a role for *Gabra6* and *Nf1* in PACAP-mediated neuroprotection. Similarly, *Il6*, *S100a5*, *Il22*, *Il1b*, *Igf1*, and *Ccl2* were highly expressed at 6 h in the PACAP-treated ischemic brain, whereas their expression level decreased at 24 h compared to the PACAP effect alone (Figure 2). On the other hand, *Fgf21*, *Pitpnc1*, and *Epha3* genes showed an increase in expression at 24 h (Figure 2). These results suggest a clear demarcation in the expression of genes to the administered PACAP38 with time, and that may be linked to the progression of ischemia itself. However, further detailed studies will be necessary to provide concrete evidence for the hypothesis.

Here, we would like to emphasize an early first study on neuroprotection by endogenous and exogenous PACAP following stroke that was performed by the Lee E Eiden’s group at the NIH (Bethesda, MD, USA). In that study the cerebrocortical transcriptional response in the MCAO model was studied in PACAP-deficient mice and in wild-type mice using a 36K cDNA microarray chip for transcriptome profiling of gene expression changes in the cortex at 1 and 24 h post-ischemia [8]. A large percentage of 142 up-regulated genes at 24 h were shown to require endogenous PACAP, and the authors suggest a more prominent role for PACAP in later response to injury than in the initial response [8]. Although 40 pmol of PACAP was used as an exogenous treatment, compared to the 1 pmol used in our present study, we looked at any similarities in gene expression changes with our present data at both 6 and 24 h post-ischemia (with or without PACAP). Only three upregulated genes and one downregulated gene were found to be common at 6 h, whereas two upregulated genes and twenty-four downregulated genes were found to be common at 24 h by Chen *et al*. [8]. The lack of similarity between the Chen et al. [8] study and ours may lie in 1) MCAO versus PMCAO and KO mice versus wild-type, 2) use of cDNA versus the whole genome microarray, and 3) different time points used (1 versus 6 h). Nevertheless, we believe that as the two studies have identified unique changes in gene expression, these data are complementary and represent the growing number of potential PACAP-regulated gene transcripts in the ischemic brain.

#### Tabulation of the differentially expressed genes

The next step was the analysis of the vast microarray data (gene lists). As we had previously reported an inventory of ischemia-related genes in the ipsilateral brain hemisphere [14], we wanted to know at first how many and what kind of genes PACAP alone influences in the brain (ipsilateral hemisphere). Using the processed data from the previous study [14] and this study on changes in gene expression > 1.5-fold for upregulated and < 0.75-fold for downregulated gene expression, at both 6- and 24-h time points, we were able to list the ischemia-related and PACAP-related genes. The first two columns of Table 1 show the results of the comparison, and it is immediately clear that PACAP influences the expression of a lower number of genes (263 to 743; PACAP-related) genome-wide over the large number of genes (622 to 2,759; ischemia-related) showing changed expression under ischemia alone. Interestingly, it was seen that the PACAP38 injection influenced a greater number of gene expressions early, that is, at 6 h post-ischemia, rather than at 24 h, suggesting that PACAP indeed has a role in regulating gene expression in the brain post-injection, a character of the neuropeptide. We next asked how many genes are influenced by PACAP under ischemia, and eliminating the ischemia alone-related genes, we could narrow down our inventory to a considerably lower number of genes (third column, Table 1). But this is again a significantly high number of genes to discuss in single study. Nonetheless, all these genes expression data are open and freely available on the GEO website at NCBI (GSE 28201 [14]; GSE 37565, this study) for the scientific community.

**Table 1:** Changes in gene expression in the ipsilateral hemisphere revealed by comparison of genes expressed under ischemia with or without pituitary adenylate cyclase-activating polypeptide (PACAP)-38 injection over saline control

**Sampling time (expression change)**	**Ischemia-related**	**PACAP-related**	**PACAP in ischemia-related**	**Common: ischemia-related and PACAP in ischemia-related**	**Common: PACAP-related and PACAP in ischemia-related**
6 h (upregulated)	1,237	435	839	171	43
6 h (downregulated)	622	743	498	31	24
24 h (upregulated)	2,759	263	831	49	30
24 h (downregulated)	2,104	271	1468	28	75

Results are presented as number of genes.

To further narrow down our search, we also looked at the common genes between two sets of data, that is, ischemia-related and PACAP in ischemia-related (fourth column, Table 1) and PACAP-related and PACAP in ischemia-related (fifth column, Table 1). The gene numbers decreased drastically to 171 and 31 (upregulated and downregulated at 6 h, respectively; Additional file 9: Table S3), and 49 and 28 (upregulated and downregulated at 24 h, respectively; Additional file 10: Table S4) for the predominantly ischemic group, and to 43 and 24 (upregulated and downregulated at 6 h, respectively; Additional file 11: Table S5), and 30 and 75 (upregulated and downregulated at 24 h, respectively; Additional file 12: Table S6) for the predominantly PACAP group. For details, see the genes listed in Additional files 9: Table S3, 10: Table S4, 11: Table S5, 12: Table S6.

#### Functional categorization of differentially expressed genes and candidate genes regulated by PACAP38 with potential links to ischemic neuroprotection

**Figure 3:**
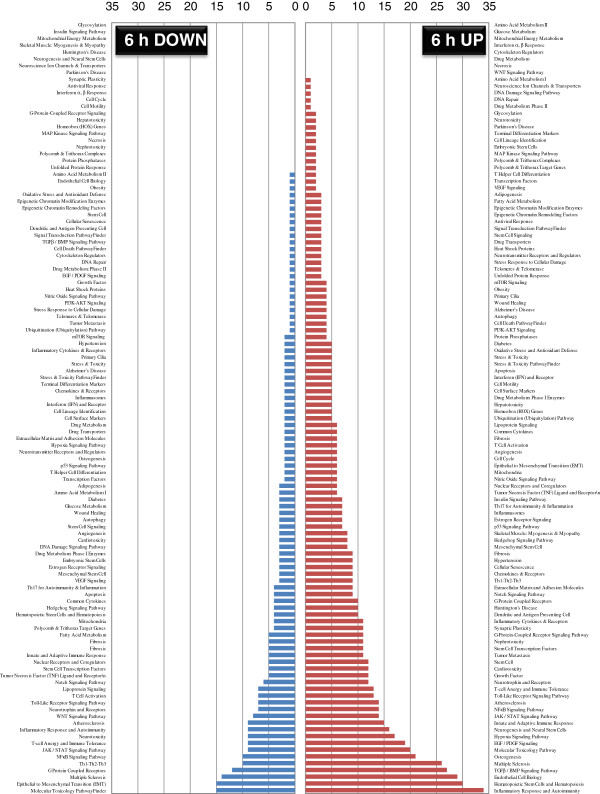
**Pathway and disease states-focused gene classification of pituitary adenylate cyclase-activating polypeptide (PACAP)- influenced genes at 6 h post-ischemia.** The up- and downregulated genes at 6 h hours after ischemia (ipsilateral hemisphere) were classified based on the available categories of more than 100 biological pathways or specific disease states in the SABiosciences PCR array list (QIAGEN; http://www.sabiosciences.com) for *Mus musculus*. The numbers in the y-axis represent the number of genes in each category, which are indicated on the x-axis.

**Figure 4:**
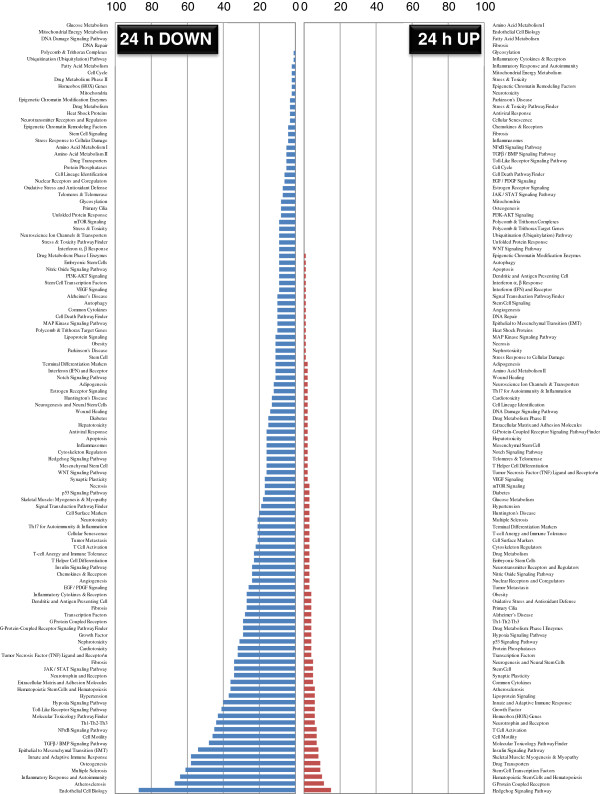
**Pathway and disease states-focused gene classification of pituitary adenylate cyclase-activating polypeptide (PACAP)-influenced genes at 24 h post-ischemia.** The up- and downregulated genes at 24 h hours after ischemia (ipsilateral hemisphere) were classified based on the available categories of more than 100 biological pathways or specific disease states the same as mentioned in Figure 3 in the SABiosciences PCR array list (QIAGEN; http://www.sabiosciences.com) for *Mus musculus*. The numbers in the y-axis represent the number of genes in each category, which are indicated on the x-axis.

To overcome the challenge of explaining the large gene dataset, we also functionally categorized the obtained gene lists (Additional files 13: Table S7, 14: Table S8, 15: Table S9 16: Table S10; 6 and 24 h up/downregulation) using the pathway- or specific disease states-focused gene classifications available on the QIAGEN website (SABiosciences; http://www.sabiosciences.com) in order to reveal the trend of predominant pathways affected by PACAP in the ischemic hemisphere at 6 and 24 h post-ischemia. Results show the distribution of genes among multiple categories (Figures 3 and 4). At 6 h after PACAP treatment, the number of upregulated gene expressions is evident in contrast to the large number of downregulated gene expression at 24 h. This indicates that PACAP influences the regulation of numerous gene pathways in the ischemic hemisphere quite early, and that might have a role in neuroprotection. Based on the listing of the gene candidates and their functional categorization, we discuss potential PACAP-regulated genes, including a novel gene in the context of a role for PACAP as a neuroprotective peptide in limiting ischemia.

#### PACAP influences early regulation/induction of interleukins

The predominant gene with annotated function influenced by PACAP38 injection was interleukin 6 (*IL6*; 4.3-fold upregulated at 6 h; Additional file 9: Table S3) upon analyzing the genes with focus on the keywords ischemia and PACAP (fourth column, Table 1). By RT-PCR, the *IL6* mRNA abundance was also found to be strongly increased under PACAP treatment at 6 h in the ischemic hemisphere over both the sham and PMCAO alone expression levels (Figure 2). On the other hand, when focusing on the genes under the keywords PACAP and ischemia (fifth column, Table 1), no interleukins were found to be upregulated (Additional files 11: Table S5 and 12: Table S6). This is because in the ischemic hemisphere at 24 h, the PACAP plus PMCAO expression level was reduced compared to the sham plus PACAP control. This suggests that interleukin is only one target of PACAP action under ischemia, and PACAP might regulate different gene sets at different stages of ischemia progression. In other words, the influence of PACAP on interleukins should logically be early rather than late, to see the neuroprotective effects of PACAP38 administration to the brain.

*IL6* is one of the most well-known and critical inflammatory cytokines in the ischemic brain [26]. Further, this result also re-confirms, and at the same time differentiates, the finding of our previous study on ischemic brain gene expression profiles [14], where *IL6* was induced to very high levels (10.35-fold and 89.23-fold upregulated at 6 and 24 hours, respectively). It has been reported that PACAP stimulates IL-6 expression from neurons and astrocytes after ischemia [12], and the neuroprotective effect of PACAP mediates the IL-6 pathway [13]. This evidence supports our findings of increased *IL6* expression in acute response after PACAP injection. Surprisingly, the *IL6* gene was not among the 49 common genes at 24 h post-treatment, suggesting that PACAP negatively influences *IL6* expression after acute response. *IL6* seems to respond and rebound after the effects of PACAP deficiency at 24 hours after injection. Using this analytical approach, we can begin to see a certain function of PACAP38 in regulating the expression of the IL inflammatory cytokines, namely, PACAP activates the differential and early induction of ILs during ischemia progression.

Further, among the numerous cytokines found to be induced in the ischemic hemisphere [14], only *IL1b*, *IL8rb*, *ILl1rap*, and *IL11* were found to be upregulated at 6, but not at 24 h (Additional files 9: Table S3 and 10: Table S4). IL-11, a member of the IL-6 family of proinflammatory cytokines, can be a potential target for PACAP-mediated neuroprotection. IL-11 also has a role in the response to oxidative stress and compensatory proliferation [27], and has recently been proposed as a candidate cytokine clinically available for cardioprotection therapy [28,29]. Another potential target is the IL-1 receptor accessory protein gene *IL1rap*. The IL-1RAP is a necessary part of the interleukin 1 receptor complex that initiates signaling events to activate the IL-1-responsive genes. Further, the *IL1rap* is also associated with IL-1 beta (IL-1b), and an early study had revealed that IL-1RAcP is necessary for centrally mediated neuroendocrine and immune responses to IL-1beta [30,31]. These reports imply that IL-11 and IL-1 pathway could connect with PACAP-induced neuroprotection after brain ischemia.

#### Novel induction of the cytotoxic and regulatory T cell molecule (Crtam) gene at 6 h

We identified *Crtam*, also called the MHC class-1-restricted T-cell-associated molecule, as a 4.2-fold upregulated gene at only 6 h (Additional file 9: Table S3). To our knowledge, this is the first report of *Crtam* gene expression by PACAP. The *Crtam* gene was initially identified as a new member of the immunoglobulin superfamily (Ig-SF) from the molecular analysis of natural killer T (NKT) cells, by Kennedy *et al*. [32]. CRTAM is also expressed by other types of activated T cells, CD4^+^ and CD8^+^. Interestingly, CRTAM maintains the T cell’s polarity, which regulates T cell activation and movement through the lymphatic system and tissues [33]. Moreover, CRTAM regulates IFN and IL-22 production [34]. In our RT-PCR results, the *IL22* mRNA expression was also strongly upregulated in PACAP administration at 6 h compared to the control (Figure 2), suggesting that PACAP affects activation and invasion of T cells in the ischemic brain. A recent review has stated that T lymphocytes are activated, infiltrated into the brain, and appear to release cytokines to contribute to the early inflammation and brain injury following an ischemic stroke [35]. PACAP-induced neuroprotection may mediate T cell activation and infiltration to regulate the acute phase of inflammation.

During the initial finding of this gene in lymphoid tissues, the *Crtam* RNA was also found in the brain as expected, but its significance has remained unclear [32]. In 2006, CRTAM was characterized and found to be highly expressed in the human cerebellum, particularly the Purkinje neurons, and where it was reported that CRTAM/Necl-2 binding may contribute to neuronal interactions [36]. Recently, the *Crtam* gene was also reported as an ethanol-responsive gene, and associated with alcohol preference in mice [37]. Although, we do not know the neuronal CRTAM function, based on the few reports available to date, it is tempting to speculate a neuroprotective role under PACAP influence in the ischemic hemisphere. Moreover, as with the induction of *IL* genes, it is of interest to note that *Crtam* is also regulated by PACAP38 at 6 but not 24 h, suggesting that PACAP effects at the molecular level can be seen within hours of administration to the brain. This result is also in accordance with our observation with the RT-PCR data that the effect of PACAP on gene expression can be divided into an early response (with potential involvement in neuroprotection) and late response (secondary effects) in the ischemic hemisphere. Nevertheless, additional experiments by functional approaches using the candidate genes are necessary to demonstrate the mechanism behind the action of PACAP.

### Two-dimensional gel electrophoresis western blotting, and immunofluorescent staining analysis identifies CRMP2 as a candidate protein factor in PACAP-influenced neuroprotection in the ischemic hemisphere

**Figure 5:**
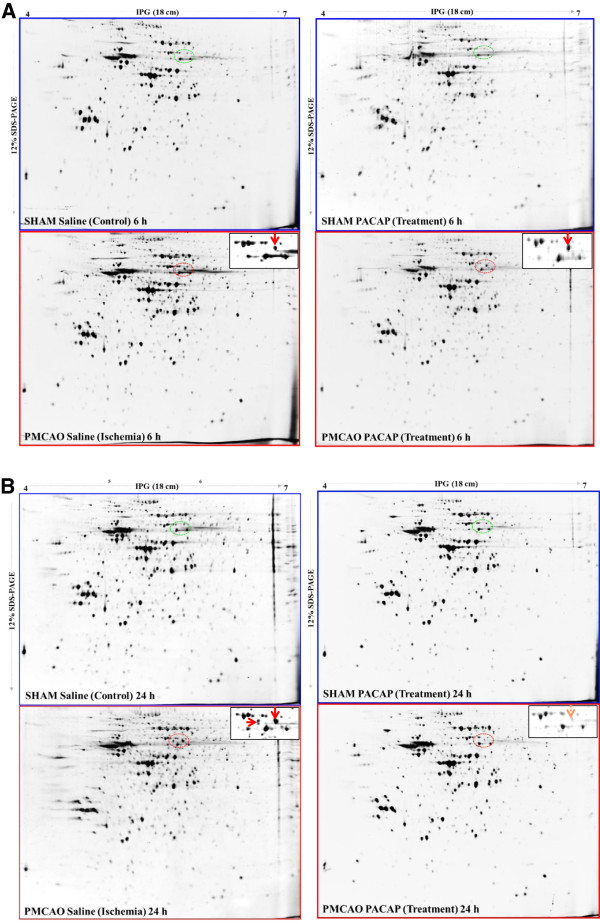
**Two-dimensional gel electrophoresis of the mouse brain.** (**A**) Total protein in the Sham (control) and PMCAO (ischemic) hemispheres were stained with Flamingo stain. (**A**) Separated proteins at 6 h after control and ischemia treatments, with (gels on right-hand side) or without PACAP38 (gels on left-hand side), respectively. (**B**) Separated proteins in the same profile as above at 24 h. Newly appearing protein/s in the ischemic hemispheres are indicated by the red dotted line circles, and the green dotted line circles represent the corresponding areas of the saline and PACAP samples. Inset: enlarged circle protein profiles. Total protein extraction, separation, staining, and image analyses procedures are detailed in Methods.

Total soluble protein profiles were first examined by one-dimensional gel electrophoresis (SDS-PAGE). Staining the separated proteins with Flamingo fluorescent stain showed no significant differences in polypeptide patterns among all the samples examined (Additional file 6: Figure S4). Therefore, we proceeded to two-dimensional analysis. Flamingo-stained two-dimensional gel protein spots clearly revealed a major and newly appeared spot with a molecular weight of approximately 60 kDa and a pI of 5.5, in the ischemic hemisphere at 6 h (lower left two-dimensional gel image, Figure 5A) over the sham saline sample; this spot was also observed in the PACAP38-treated ischemic brain (lower right two-dimensional gel, Figure 5A) over the sham PACAP sample. Interestingly, the same spot was also observed at a much stronger intensity (more than 2-fold) in the ischemic hemisphere at 24 h (lower left two dimensional gel image, Figure 5B) but not in the sham saline sample. Two additional minor spots appeared at approximately the same molecular weight range but with a slightly acidic pI (isoelectric point). Surprisingly, and in contrast to the 6 h results, the major and minor spots were not found in the PACAP38-treated brain (lower right two-dimensional gel image, Figure 5B) over the sham PACAP sample. To identify the protein in these newly appearing spots, we excised these three spots from the stained gels and after in-gel trypsin digestion analyzed the peptides by MALDI-TOF-MS, as described in Methods. The MASCOT search revealed the major abundant protein to be a dihydropyrimidinase-related protein 2 (DPYL2_MOUSE) or collapsin response mediator protein 2 (CRMP2_MOUSE). No significant data (protein IDs) could be obtained for the two protein spots with the lowest abundance appearing exclusively at 24 h post ischemia. The CRMP2 protein, as it more popularly known, is involved in axonal growth and neuronal differentiation [17]. Although there is no information to date on the effects of PACAP on the CRMP2 protein, a few studies have already shown that the CRMP2 protein is induced in the brain after focal cerebral ischemia, in old mouse brains, and in the cerebral cortex of rats [38-40].

**Figure 6:**
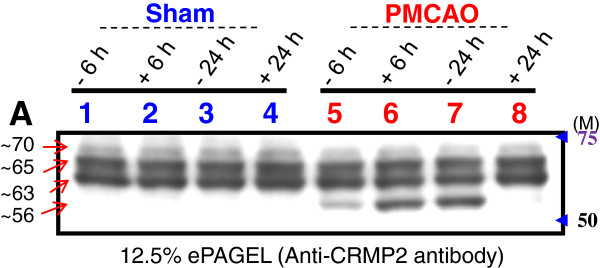
**Western blot analysis of the CRMP2 protein cross-reacting proteins in sham control and permanent middle cerebral artery occlusion (PMCAO) with or without pituitary adenylate cyclase-activating polypeptide (PACAP) treatment.** Proteins cross-reacting with the anti-CRMP2 protein antibody are visible as three constitutively present proteins (approximately 70, 65, and 63 kDa size) in all samples. The approximately 56 kDa cross-reacting protein is seen only in the ischemic hemisphere (PMCAO). Lanes 1, 2 and 5, 6 are hemispheres dissected out at 6 h after control and ischemia treatments respectively. The symbol – (minus) indicates without PACAP38, that is, 1 μL of 0.9% saline, whereas + (plus) indicates with 1 pmol (1 μL) of PACAP38 injection. Total protein extraction, separation, Western blotting and image analyses procedures are detailed in Methods. Total protein in the Sham (control) and PMCAO (ischemic) hemispheres were stained with Flamingo stain as shown in Additional file 6: Figure S4.

We next examined by western blot analysis the CRMP2 protein profile on SDS-PAGE. Using a specific CRMP2 protein antibody three main bands of approximately 70, 65, and 63 kDa were detected (Figure 6). No significant change in protein expression was seen among the sham control and PMCAO samples, with or without PACAP38 treatment. However, in the PMCAO samples only, at an approximate molecular weight of 56 kDa, a cross-reacting protein band was seen. At 6 h post-ischemia, the 56 kDa protein was increased in abundance over the minus-PACAP sample. At 24 h post-PACAP treatment the 56 kDa protein band was found at very low levels. This result matches the induction profile of the major protein spot seen on the two-dimensional gels. Further, using two anti-phosphorylated CRMP2 proteins antibodies, approximately 70 and 65 kDa and 70 kDa cross-reacting bands were observed. However, no significant change in abundance of protein bands was observed (Additional file 17: Figure S7). No specific band at the 56 kDa molecular weight was seen in the PMCAO group. These data rule out the possibility of protein phosphorylation as a post-translational modification for the appearance of the 56 kDa cross-reacting protein band.

**Figure 7:**
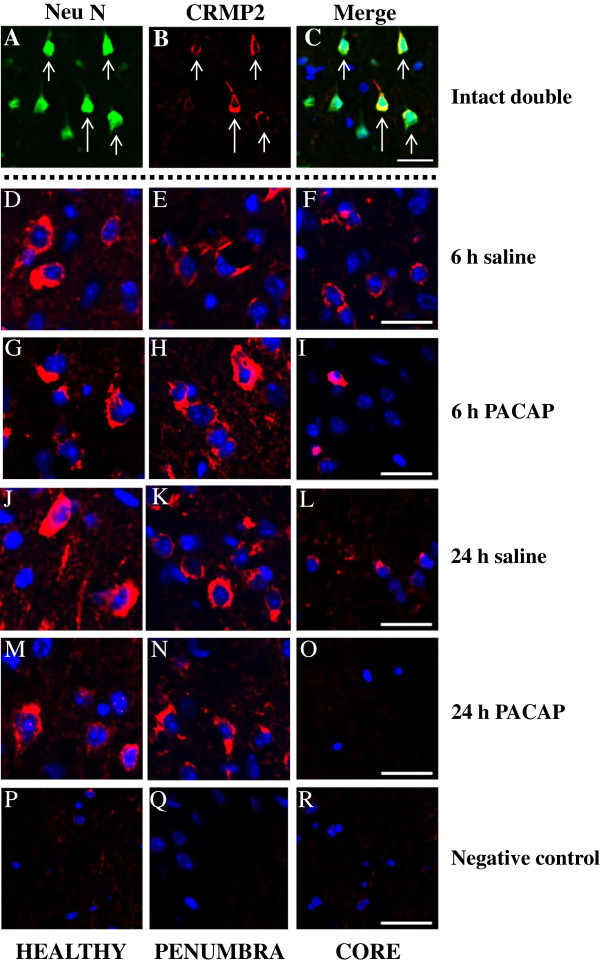
**Immunofluorescent staining.** Neurons express CRMP2 at 6 and 24 h after permanent middle cerebral artery occlusion (PMCAO). A,B,C: triple immunofluorescence staining for CRMP2, NeuN and 4′, 6-diamidine-2-phenylindole dihydrochloride (DAPI); the merged picture displays expression of CRMP2 by NeuN positive neuron but not DAPI-positive neurons. D,G,J,M,P: healthy; E,H,K,N,Q: penumbra; and F,I,L,O,R: core. D-R: double immunofluorescence staining for CRMP2, and DAPI; the merged picture shows mutually exclusive expression of neuron. The merged picture shows membranous localization of CRMP2 surrounding DAPI-positive nuclei. All images were captured in the ipsilateral hippocampus. Intact (double): A, B, C; 6 h saline: D, E, F; 6-h pituitary adenylate cyclase-activating polypeptide (PACAP): G, H, I; 24 h saline: J, K, L; 24 h PACAP: M, N, O; negative control: P, Q, R. Green - NeuN, Red - CRMP2, and Blue - DAPI. Scale bar: 20 μm. The sections were prepared as described in Methods; see also Additional file 8: Figure S6.

Immunofluorescent staining using anti-CRMP2 antibody revealed that the CRMP2 protein is localized to the cytoplasm in neuronal cells as is evident from the healthy region of the ipsilateral hemisphere (Figure 7). Interestingly, in the penumbra, the CRMP2 protein appears to be more abundant in the 6-h PACAP group. In the core region, the CRMP2 protein is reduced in abundance, in particular under PACAP38 treatment, especially prominent by almost no presence of CRMP2 at 24 h after PACAP treatment to the ischemic brain. Comparing these three data, it can be stated that 1) two-dimensional gel electrophoresis and western blot analysis confirmed the presence of a newly appearing 56 kDa protein, 2) PACAP treatment influences its induction at 6 h post-treatment in the ischemic hemisphere, and 3) a very low level of the CRMP2 protein on two-dimensional gel at 24 h post-ischemia under PACAP treatment correlates well with the results seen from western blot analysis data and immunofluorescent staining of the core region. In other words, our results show that PACAP treatment affects the expression of CRMP2 protein, but how PACAP and CRMP2 are involved in neuroprotection of the brain remains to be answered.

## Conclusions

To our knowledge the omics-based study demonstrates significant change in gene and protein expressions after PACAP38 injection in the ischemic brain. Results presented in the study highlight the usefulness of global gene expression profiling in searching for changes in gene expression, and delineating the molecular events in a defined experimental PMCAO model. Furthermore, the proteomics approach proved to be critical in identifying the CRMP2 protein, which was surprisingly the most abundant and newly appearing protein spot on the two-dimensional gel. Post-confirmation of the two-dimensional gel spot by MS and western blot analyses, immuno-double staining revealed the possibility of CRMP2 as a controlling factor in PACAP-regulated control of ischemic neuroprotection. Forward or reverse genetic approaches involving certain target genes, including the most promising candidate CRMP2 protein, will be necessary to increase our understanding of the functional role of various genes in response to an ischemic injury and to clarify the precise mechanism behind PACAP38-induced neuroprotection. Furthermore, to further enhance our understanding of the functional roles of the many identified gene and protein candidates, future work such as utilizing the integrated pathway analysis (IPA) software for predicting the possible pathways and underlying mechanisms of PACAP-mediated molecular changes, and experiments, especially with the PACAP knockout mice, will be essential. Finally, the numerous specific PACAP target transcripts that were unannotated have not been discussed here, and will be also a target for future studies.

## Abbreviations

CAN: Acetonitrile; CNS: Central nervous system; CRMP2: Collapsin response mediator protein-2; Crtam: Regulatory T cell molecule; DAPI: 4′ 6-diamidine-2-phenylindole dihydrochloride; DPYL2: Dihydropyrimidinase-related protein 2; IL: Interleukin; MALDI-TOF-MS: Matrix-assisted laser desorption/ionization-time of flight-mass spectrometry; MS: Mass spectrometry; NG: Neurological grade; PACAP: Pituitary adenylate cyclase-activating polypeptide; PB: Phosphate buffer; PBS: Phosphate-buffered saline; PMCAO: Permanent middle cerebral artery occlusion; PVDF: Polyvinyldifluoride; TFA: Trifluoroacetic acid; VIP: Vasoactive intestinal polypeptide.

## Competing interests

The authors declare they have no competing interests.

## Authors' contributions

MH, TN, RR, JS, and SS discussed and designed the study plan. MH and TN performed the animal experiments. MH, TN, RR, JS, and TA designed and performed the genomics and proteomics experiments. MH, TN, RR, JS, and TO analyzed the data. MH and RR wrote the paper. MH, TN, RR, TT, KT, and SS checked, revised and finalized the paper. All authors read and approved the final manuscript.

## Supplementary Material

Additional file 1 **Table S1.** The number of mice used for this experiment. A total of 23 mice were prepared, and 3 each were selected randomly based on neurological grade (NG). Mice with score 0 and 3 were not selected for sampling the brains. Click here for file

Additional file 2 **Table S2.** Primer combinations used for RT-PCR. Click here for file

Additional file 3 **Figure S1.** Preparation of LB-TT. Click here for file

Additional file 4 **Figure S2.** ProteoExtract Protein Precipitation Kit Protocol (Illustrated). Click here for file

Additional file 5 **Figure S3.** Two-dimensional gel electrophoresis. (2-DGE) Click here for file

Additional file 6 **Figure S4.** One-dimensional gel electrophoresis (SDS-PAGE). Click here for file

Additional file 7 **Figure S5.** Dissected Brain Storage and Sectioning Protocol (Illustrated). Click here for file

Additional file 8 **Figure S6.** Dissected Regions as Ischemic core, Penumbra and Healthy of the Ipsilateral Hemisphere (Illustrated). Click here for file

Additional file 9 **Table S3.** Effect of PACAP 38 injection on expression of genes at 6 h with a focus on ischemia and PACAP. Click here for file

Additional file 10 **Table S4.** Effect of PACAP 38 injection on expression of genes at 24 h with a focus on ischemia and PACAP. Click here for file

Additional file 11 **Table S5.** Effect of PACAP 38 injection on expression of genes at 6 h with a focus on PACAP and ischemia. Click here for file

Additional file 12 **Table S6.** Effect of PACAP 38 injection on expression of genes at 24 h with a focus on PACAP and ischemia. Click here for file

Additional file 13 **Table S7.** The functionally categorized (pathway- or specific disease states-focused gene classifications) upregulated genes at 6 h. Click here for file

Additional file 14 **Table S8.** The functionally categorized (pathway- or specific disease states-focused gene classifications) downregulated genes at 6 h. Click here for file

Additional file 15 **Table S9.** The functionally categorized (pathway- or specific disease states-focused gene classifications) upregulated genes at 24 h. Click here for file

Additional file 16 **Table S10.** The functionally categorized (pathwayor specific disease states-focused gene classifications) downregulated genes at 24 h. Click here for file

Additional file 17 **Figure S7.** Western blot analysis of the anti-phosphorylated CRMP2 proteins cross-reacting proteins. Click here for file
