# Correction: Transcriptomics and proteomics analyses of the PACAP38 influenced ischemic brain in permanent middle cerebral artery occlusion model mice

**DOI:** 10.1186/1742-2094-10-18

**Published:** 2013-01-31

**Authors:** Motohide Hori, Tomoya Nakamachi, Randeep Rakwal, Junko Shibato, Tetsuo Ogawa, Toshihiro Aiuchi, Tatsuaki Tsuruyama, Keiji Tamaki, Seiji Shioda

**Affiliations:** 1 Department of Forensic Medicine and Molecular Pathology, School of Medicine, Kyoto University, 606-8315, Kyoto, Japan; 2 Department of Anatomy I, School of Medicine, Showa University, 1-5-8 Hatanodai, 142-8555, Shinagawa, Tokyo, Japan; 3 Department of Center for Biotechnology, Showa University, 1-5-8 Hatanodai, Shinagawa, Tokyo 142-8555, Japan; 4 Graduate School of Life and Environmental Sciences, University of Tsukuba, 305-8572, Tsukuba, Japan

## 

The Figure Two (Figure 1 here), X-axis description of each sample was inverted in the original publication [1].

**Figure 1:**
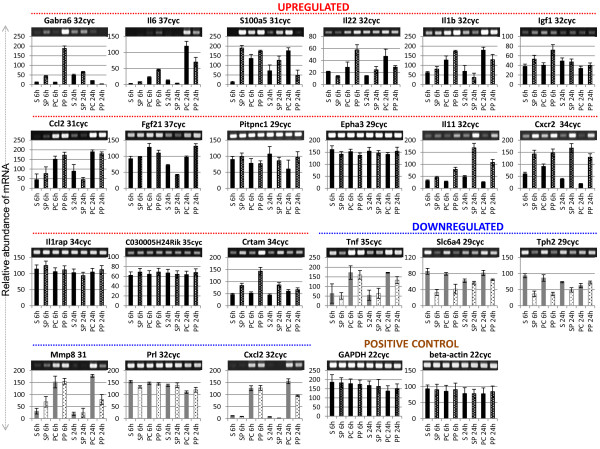
**The mRNA expression profiles of differentially expressed genes.** Both the upregulated (**A**) and downregulated (**B**) genes were selected randomly. Gel images on top show the polymerase chain reaction (PCR) product bands stained with ethidium bromide; the band intensities are also presented graphically below for clarity. Lane numbers 1 to 8 indicate sham control (lanes 1, 2, 5, and 6) and permanent middle cerebral artery occlusion (PMCAO) treatment (lanes 3, 4, 7, and 8), respectively. P indicates pituitary adenylate cyclase-activating polypeptide (PACAP) treatment; C is the control (minus PACAP). *GAPDH* and *beta-actin* genes were used a positive control (**C**). Semi-quantitative RT-PCR was performed as described in Methods, and the specific 3’-UTR primers are detailed in Additional file 2: Table S2.

With reference to corrected Figure Two (Figure 1 here), we have the following revised text.

On Page 9, left column: lines 19-24 should read as -

“Similarly, *Il6*, *S100a5*, *Il22*, *Il1b*, *Igf1*, and *Ccl2* were highly expressed at 6 h in the PACAP-treated ischemic brain, whereas their expression level decreased at 24 h compared to the PMCAO effect alone (Figure Two (Figure 1 here)). *Fgf21*, *Pitpnc1*, and *Epha3* genes showed an increase in expression at 24 h over PMCAO alone (Figure Two (Figure 1 here)).”

On Page 11, right column: lines 16-19 should read as -

“In the ischemic hemisphere at 24 h, the PACAP plus PMCAO expression level of *Il6* was also reduced compared to the PMCAO plus saline control.”

We regret any inconvenience that this inaccuracy in Figure Two (Figure 1 here) and therein the figure legend, which could not be properly corrected at the proof stage, in the originally published manuscript might have caused.
