# Supplementary material for: Transcriptomics and proteomics analyses of the PACAP38 influenced ischemic brain in permanent middle cerebral artery occlusion model mice
Source: J Neuroinflammation. 2012 Nov 23;9:256. doi: 10.1186/1742-2094-9-256 (PMC3526409; doi:10.1186/1742-2094-9-256)
Supplement: Additional file 3 — Figure S1. Preparation of LB-TT. [file 1742-2094-9-256-S3.pptx]

## Slide 1
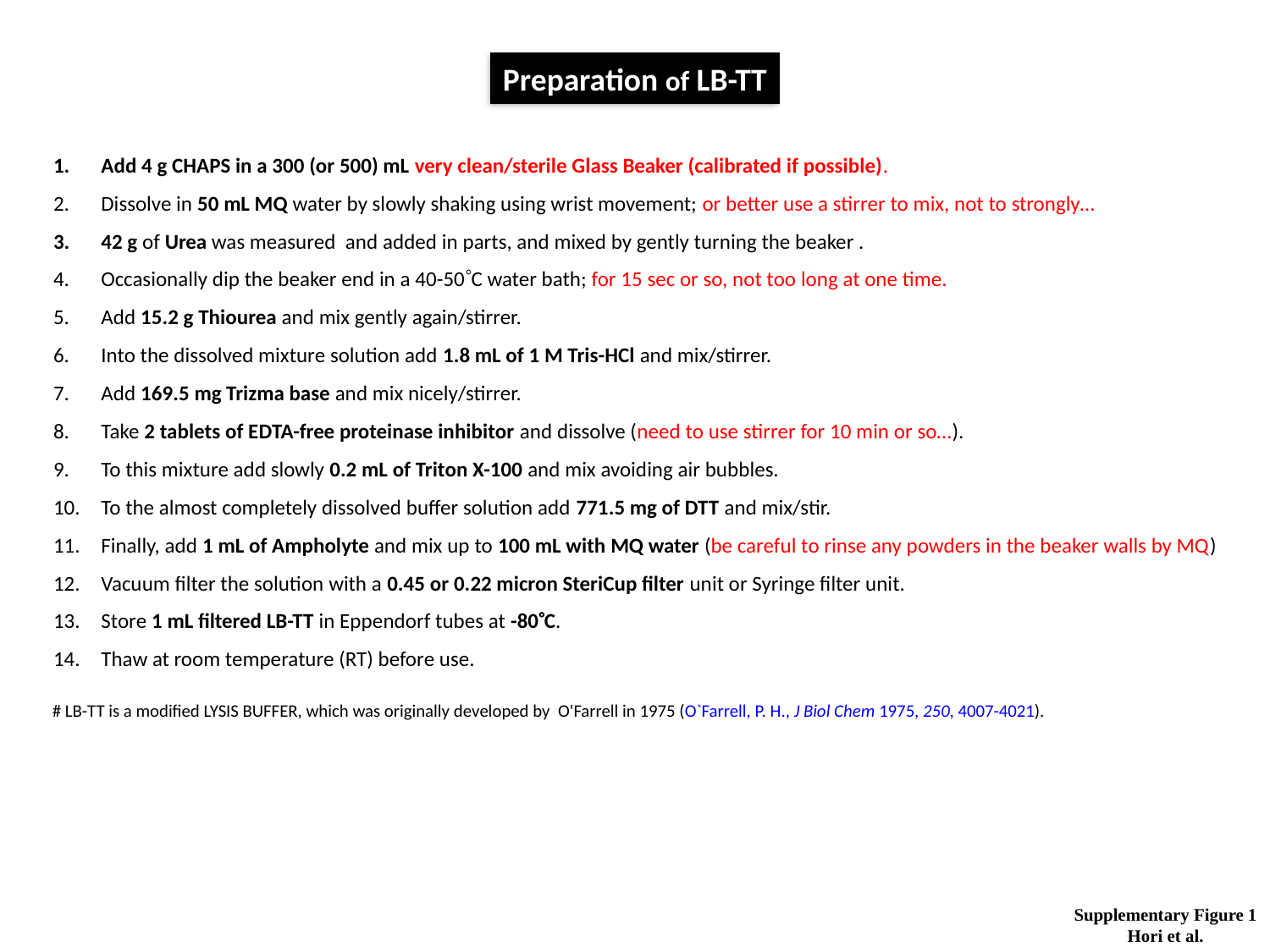

Preparation of LB-TT
Add 4 g CHAPS in a 300 (or 500) mL very clean/sterile Glass Beaker (calibrated if possible).
Dissolve in 50 mL MQ water by slowly shaking using wrist movement; or better use a stirrer to mix, not to strongly…
42 g of Urea was measured and added in parts, and mixed by gently turning the beaker .
Occasionally dip the beaker end in a 40-50°C water bath; for 15 sec or so, not too long at one time.
Add 15.2 g Thiourea and mix gently again/stirrer.
Into the dissolved mixture solution add 1.8 mL of 1 M Tris-HCl and mix/stirrer.
Add 169.5 mg Trizma base and mix nicely/stirrer.
Take 2 tablets of EDTA-free proteinase inhibitor and dissolve (need to use stirrer for 10 min or so…).
To this mixture add slowly 0.2 mL of Triton X-100 and mix avoiding air bubbles.
To the almost completely dissolved buffer solution add 771.5 mg of DTT and mix/stir.
Finally, add 1 mL of Ampholyte and mix up to 100 mL with MQ water (be careful to rinse any powders in the beaker walls by MQ)
Vacuum filter the solution with a 0.45 or 0.22 micron SteriCup filter unit or Syringe filter unit.
Store 1 mL filtered LB-TT in Eppendorf tubes at -80°C.
Thaw at room temperature (RT) before use.
# LB-TT is a modified LYSIS BUFFER, which was originally developed by O'Farrell in 1975 (O`Farrell, P. H., J Biol Chem 1975, 250, 4007-4021).
Supplementary Figure 1
Hori et al.
