# Supplementary material for: Transcriptomics and proteomics analyses of the PACAP38 influenced ischemic brain in permanent middle cerebral artery occlusion model mice
Source: J Neuroinflammation. 2012 Nov 23;9:256. doi: 10.1186/1742-2094-9-256 (PMC3526409; doi:10.1186/1742-2094-9-256)
Supplement: Additional file 4 — Figure S2. ProteoExtract Protein Precipitation Kit Protocol (Illustrated). [file 1742-2094-9-256-S4.pptx]

## Slide 1
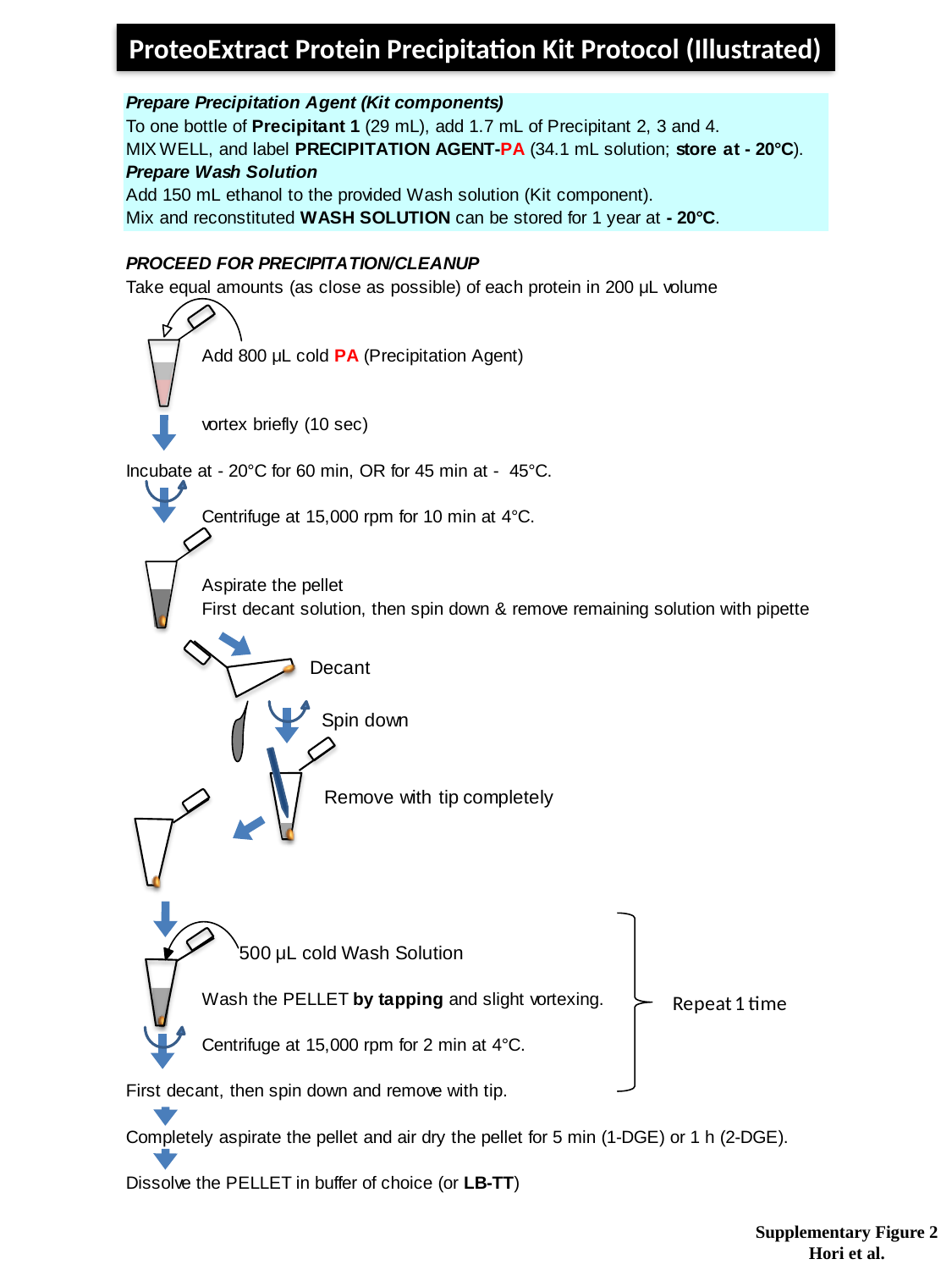

ProteoExtract Protein Precipitation Kit Protocol (Illustrated)
Supplementary Figure 2
Hori et al.
