# Supplementary material for: Transcriptomics and proteomics analyses of the PACAP38 influenced ischemic brain in permanent middle cerebral artery occlusion model mice
Source: J Neuroinflammation. 2012 Nov 23;9:256. doi: 10.1186/1742-2094-9-256 (PMC3526409; doi:10.1186/1742-2094-9-256)
Supplement: Additional file 5 — Figure S3. Two-dimensional gel electrophoresis. (2-DGE) [file 1742-2094-9-256-S5.pptx]

## Slide 1
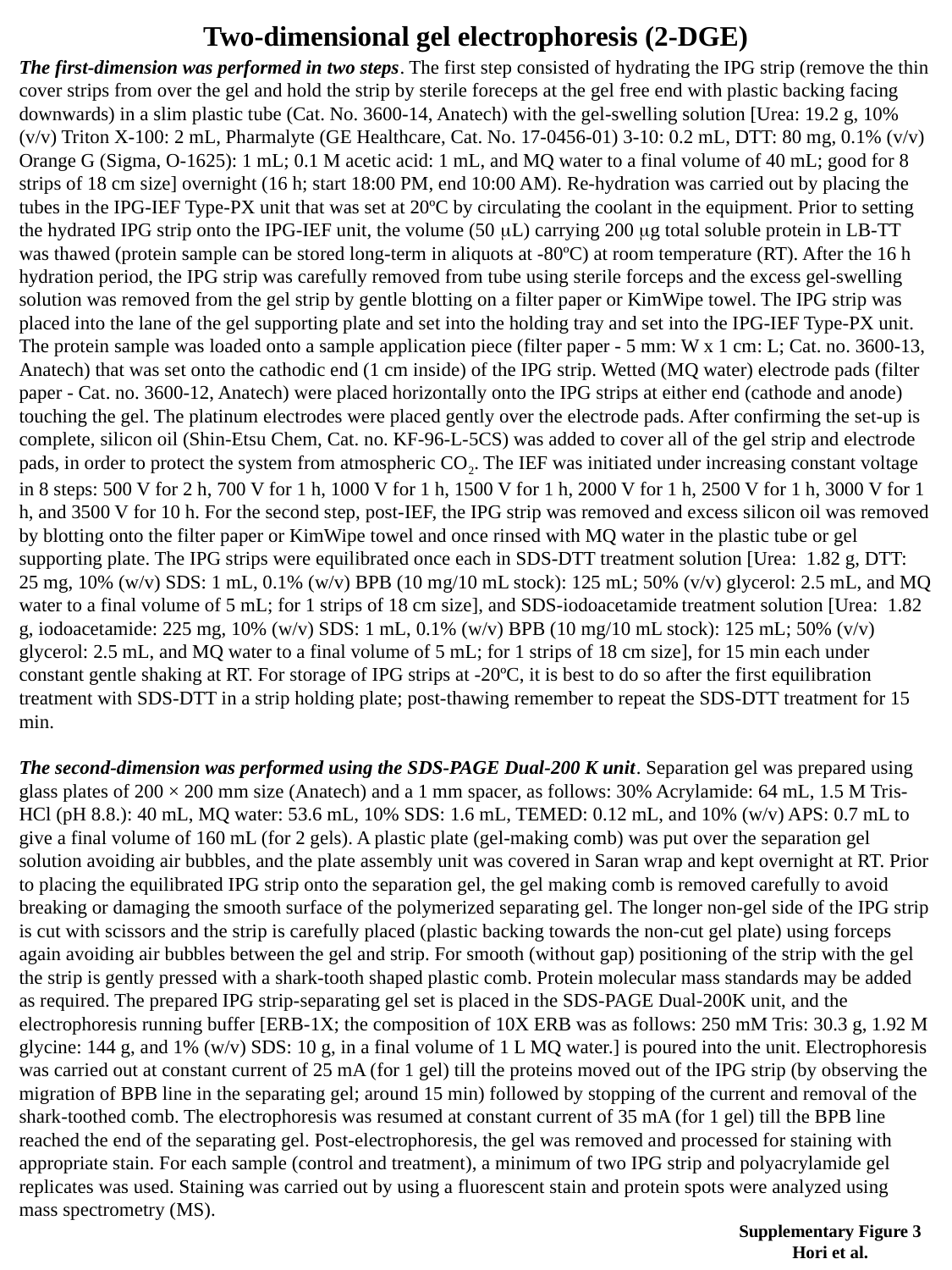

Two-dimensional gel electrophoresis (2-DGE)
The first-dimension was performed in two steps. The first step consisted of hydrating the IPG strip (remove the thin cover strips from over the gel and hold the strip by sterile foreceps at the gel free end with plastic backing facing downwards) in a slim plastic tube (Cat. No. 3600-14, Anatech) with the gel-swelling solution [Urea: 19.2 g, 10% (v/v) Triton X-100: 2 mL, Pharmalyte (GE Healthcare, Cat. No. 17-0456-01) 3-10: 0.2 mL, DTT: 80 mg, 0.1% (v/v) Orange G (Sigma, O-1625): 1 mL; 0.1 M acetic acid: 1 mL, and MQ water to a final volume of 40 mL; good for 8 strips of 18 cm size] overnight (16 h; start 18:00 PM, end 10:00 AM). Re-hydration was carried out by placing the tubes in the IPG-IEF Type-PX unit that was set at 20ºC by circulating the coolant in the equipment. Prior to setting the hydrated IPG strip onto the IPG-IEF unit, the volume (50 mL) carrying 200 mg total soluble protein in LB-TT was thawed (protein sample can be stored long-term in aliquots at -80ºC) at room temperature (RT). After the 16 h hydration period, the IPG strip was carefully removed from tube using sterile forceps and the excess gel-swelling solution was removed from the gel strip by gentle blotting on a filter paper or KimWipe towel. The IPG strip was placed into the lane of the gel supporting plate and set into the holding tray and set into the IPG-IEF Type-PX unit. The protein sample was loaded onto a sample application piece (filter paper - 5 mm: W x 1 cm: L; Cat. no. 3600-13, Anatech) that was set onto the cathodic end (1 cm inside) of the IPG strip. Wetted (MQ water) electrode pads (filter paper - Cat. no. 3600-12, Anatech) were placed horizontally onto the IPG strips at either end (cathode and anode) touching the gel. The platinum electrodes were placed gently over the electrode pads. After confirming the set-up is complete, silicon oil (Shin-Etsu Chem, Cat. no. KF-96-L-5CS) was added to cover all of the gel strip and electrode pads, in order to protect the system from atmospheric CO2. The IEF was initiated under increasing constant voltage in 8 steps: 500 V for 2 h, 700 V for 1 h, 1000 V for 1 h, 1500 V for 1 h, 2000 V for 1 h, 2500 V for 1 h, 3000 V for 1 h, and 3500 V for 10 h. For the second step, post-IEF, the IPG strip was removed and excess silicon oil was removed by blotting onto the filter paper or KimWipe towel and once rinsed with MQ water in the plastic tube or gel supporting plate. The IPG strips were equilibrated once each in SDS-DTT treatment solution [Urea: 1.82 g, DTT: 25 mg, 10% (w/v) SDS: 1 mL, 0.1% (w/v) BPB (10 mg/10 mL stock): 125 mL; 50% (v/v) glycerol: 2.5 mL, and MQ water to a final volume of 5 mL; for 1 strips of 18 cm size], and SDS-iodoacetamide treatment solution [Urea: 1.82 g, iodoacetamide: 225 mg, 10% (w/v) SDS: 1 mL, 0.1% (w/v) BPB (10 mg/10 mL stock): 125 mL; 50% (v/v) glycerol: 2.5 mL, and MQ water to a final volume of 5 mL; for 1 strips of 18 cm size], for 15 min each under constant gentle shaking at RT. For storage of IPG strips at -20ºC, it is best to do so after the first equilibration treatment with SDS-DTT in a strip holding plate; post-thawing remember to repeat the SDS-DTT treatment for 15 min.
The second-dimension was performed using the SDS-PAGE Dual-200 K unit. Separation gel was prepared using glass plates of 200 × 200 mm size (Anatech) and a 1 mm spacer, as follows: 30% Acrylamide: 64 mL, 1.5 M Tris-HCl (pH 8.8.): 40 mL, MQ water: 53.6 mL, 10% SDS: 1.6 mL, TEMED: 0.12 mL, and 10% (w/v) APS: 0.7 mL to give a final volume of 160 mL (for 2 gels). A plastic plate (gel-making comb) was put over the separation gel solution avoiding air bubbles, and the plate assembly unit was covered in Saran wrap and kept overnight at RT. Prior to placing the equilibrated IPG strip onto the separation gel, the gel making comb is removed carefully to avoid breaking or damaging the smooth surface of the polymerized separating gel. The longer non-gel side of the IPG strip is cut with scissors and the strip is carefully placed (plastic backing towards the non-cut gel plate) using forceps again avoiding air bubbles between the gel and strip. For smooth (without gap) positioning of the strip with the gel the strip is gently pressed with a shark-tooth shaped plastic comb. Protein molecular mass standards may be added as required. The prepared IPG strip-separating gel set is placed in the SDS-PAGE Dual-200K unit, and the electrophoresis running buffer [ERB-1X; the composition of 10X ERB was as follows: 250 mM Tris: 30.3 g, 1.92 M glycine: 144 g, and 1% (w/v) SDS: 10 g, in a final volume of 1 L MQ water.] is poured into the unit. Electrophoresis was carried out at constant current of 25 mA (for 1 gel) till the proteins moved out of the IPG strip (by observing the migration of BPB line in the separating gel; around 15 min) followed by stopping of the current and removal of the shark-toothed comb. The electrophoresis was resumed at constant current of 35 mA (for 1 gel) till the BPB line reached the end of the separating gel. Post-electrophoresis, the gel was removed and processed for staining with appropriate stain. For each sample (control and treatment), a minimum of two IPG strip and polyacrylamide gel replicates was used. Staining was carried out by using a fluorescent stain and protein spots were analyzed using mass spectrometry (MS).
Supplementary Figure 3
Hori et al.
