# Supplementary material for: Transcriptomics and proteomics analyses of the PACAP38 influenced ischemic brain in permanent middle cerebral artery occlusion model mice
Source: J Neuroinflammation. 2012 Nov 23;9:256. doi: 10.1186/1742-2094-9-256 (PMC3526409; doi:10.1186/1742-2094-9-256)
Supplement: Additional file 6 — Figure S4. One-dimensional gel electrophoresis (SDS-PAGE). [file 1742-2094-9-256-S6.pptx]

## Slide 1
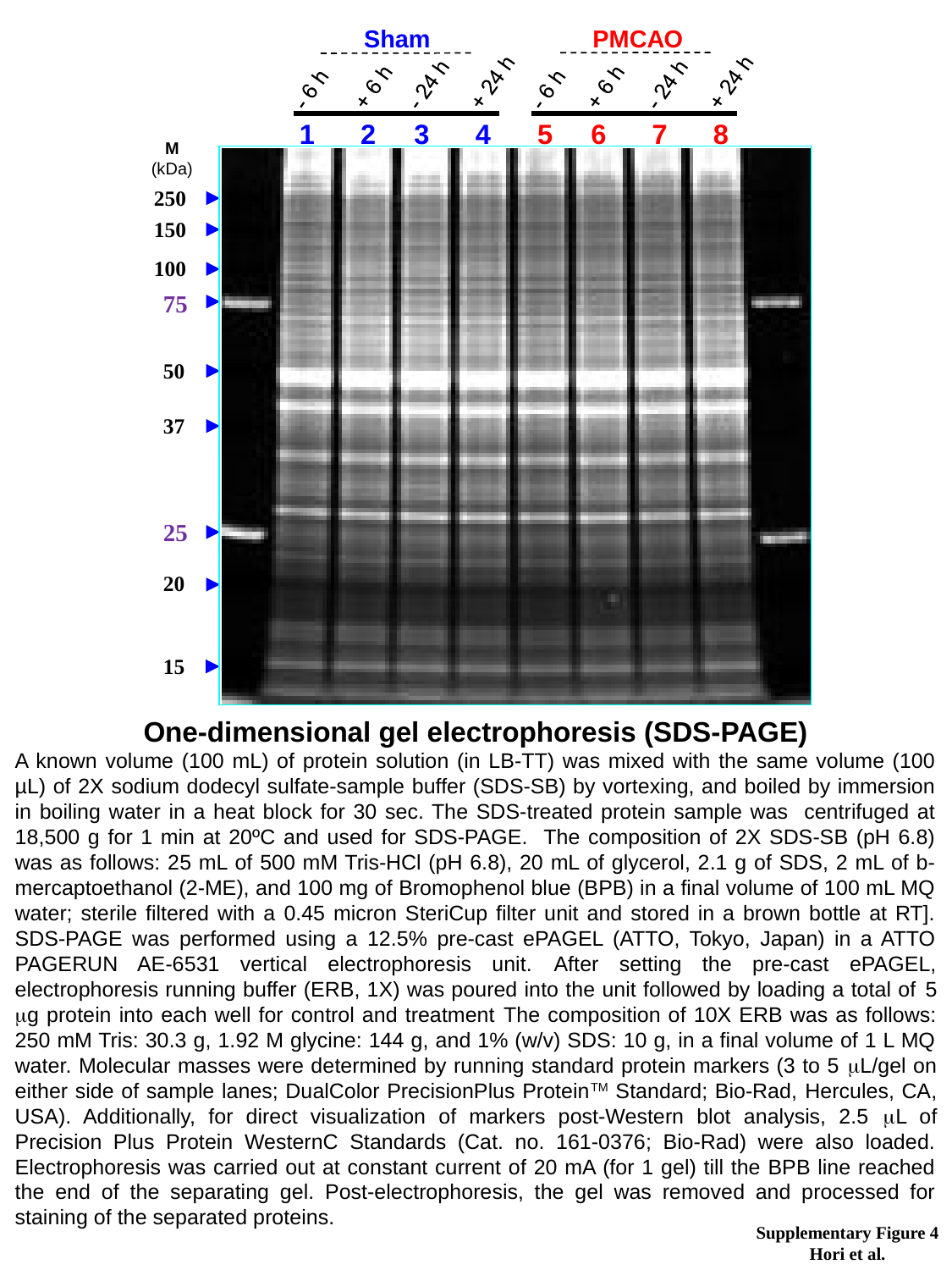

One-dimensional gel electrophoresis (SDS-PAGE)
A known volume (100 mL) of protein solution (in LB-TT) was mixed with the same volume (100 µL) of 2X sodium dodecyl sulfate-sample buffer (SDS-SB) by vortexing, and boiled by immersion in boiling water in a heat block for 30 sec. The SDS-treated protein sample was centrifuged at 18,500 g for 1 min at 20ºC and used for SDS-PAGE. The composition of 2X SDS-SB (pH 6.8) was as follows: 25 mL of 500 mM Tris-HCl (pH 6.8), 20 mL of glycerol, 2.1 g of SDS, 2 mL of b-mercaptoethanol (2-ME), and 100 mg of Bromophenol blue (BPB) in a final volume of 100 mL MQ water; sterile filtered with a 0.45 micron SteriCup filter unit and stored in a brown bottle at RT]. SDS-PAGE was performed using a 12.5% pre-cast ePAGEL (ATTO, Tokyo, Japan) in a ATTO PAGERUN AE-6531 vertical electrophoresis unit. After setting the pre-cast ePAGEL, electrophoresis running buffer (ERB, 1X) was poured into the unit followed by loading a total of 5 mg protein into each well for control and treatment The composition of 10X ERB was as follows: 250 mM Tris: 30.3 g, 1.92 M glycine: 144 g, and 1% (w/v) SDS: 10 g, in a final volume of 1 L MQ water. Molecular masses were determined by running standard protein markers (3 to 5 mL/gel on either side of sample lanes; DualColor PrecisionPlus ProteinTM Standard; Bio-Rad, Hercules, CA, USA). Additionally, for direct visualization of markers post-Western blot analysis, 2.5 mL of Precision Plus Protein WesternC Standards (Cat. no. 161-0376; Bio-Rad) were also loaded. Electrophoresis was carried out at constant current of 20 mA (for 1 gel) till the BPB line reached the end of the separating gel. Post-electrophoresis, the gel was removed and processed for staining of the separated proteins.
Supplementary Figure 4
Hori et al.
