# Supplementary material for: Transcriptomics and proteomics analyses of the PACAP38 influenced ischemic brain in permanent middle cerebral artery occlusion model mice
Source: J Neuroinflammation. 2012 Nov 23;9:256. doi: 10.1186/1742-2094-9-256 (PMC3526409; doi:10.1186/1742-2094-9-256)
Supplement: Additional file 7 — Figure S5. Dissected Brain Storage and Sectioning Protocol (Illustrated). [file 1742-2094-9-256-S7.pptx]

## Slide 1
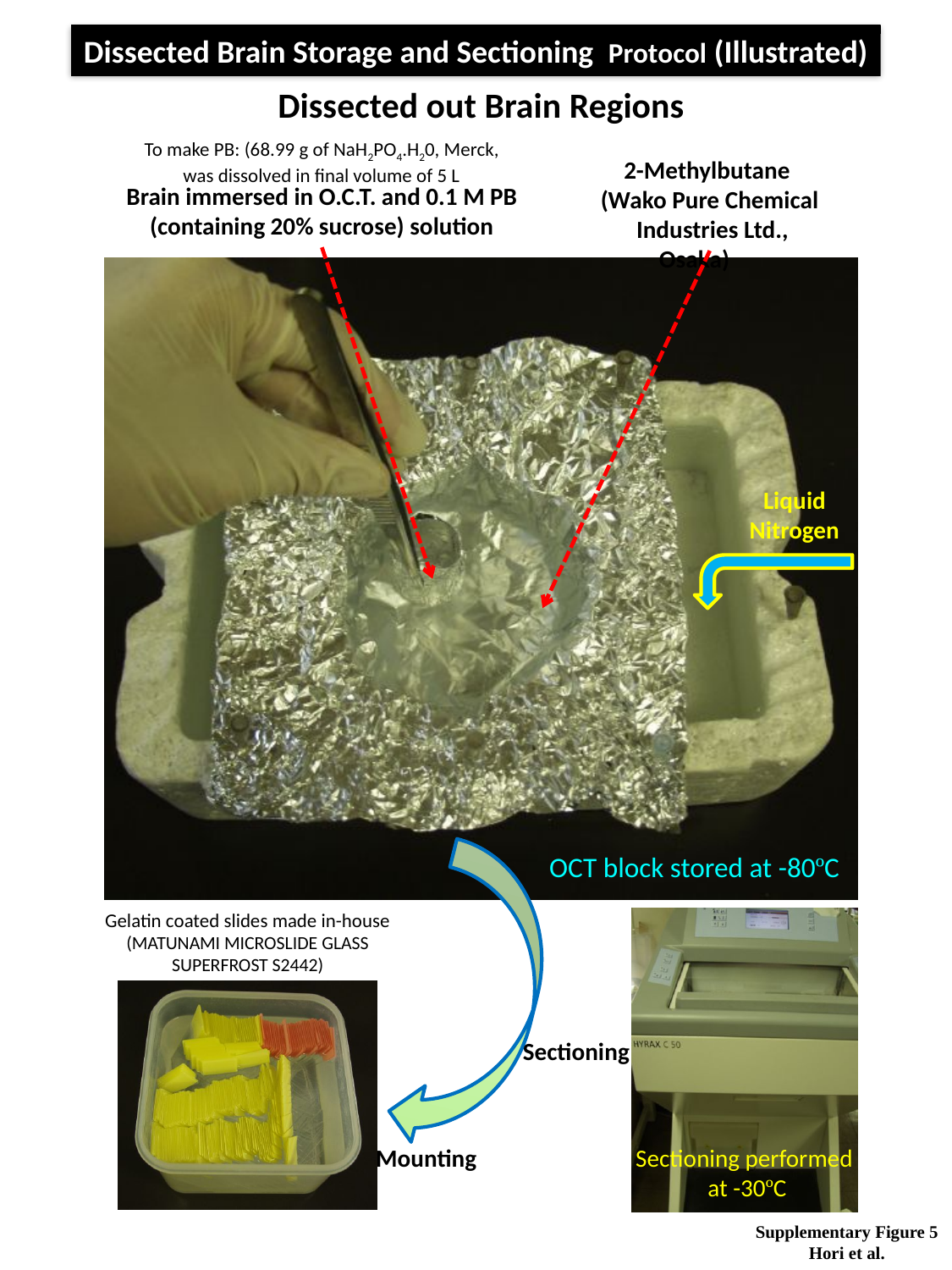

Dissected Brain Storage and Sectioning Protocol (Illustrated)
Dissected out Brain Regions
To make PB: (68.99 g of NaH2PO4.H20, Merck, was dissolved in final volume of 5 L
2-Methylbutane
(Wako Pure Chemical
 Industries Ltd., Osaka)
Brain immersed in O.C.T. and 0.1 M PB (containing 20% sucrose) solution
Liquid
Nitrogen
OCT block stored at -80ºC
Gelatin coated slides made in-house
(MATUNAMI MICROSLIDE GLASS SUPERFROST S2442)
Sectioning
Mounting
Sectioning performed
at -30ºC
Supplementary Figure 5
Hori et al.
