# Supplementary material for: Transcriptomics and proteomics analyses of the PACAP38 influenced ischemic brain in permanent middle cerebral artery occlusion model mice
Source: J Neuroinflammation. 2012 Nov 23;9:256. doi: 10.1186/1742-2094-9-256 (PMC3526409; doi:10.1186/1742-2094-9-256)
Supplement: Additional file 8 — Figure S6. Dissected Regions as Ischemic core, Penumbra and Healthy of the Ipsilateral Hemisphere (Illustrated). [file 1742-2094-9-256-S8.pptx]

## Slide 1
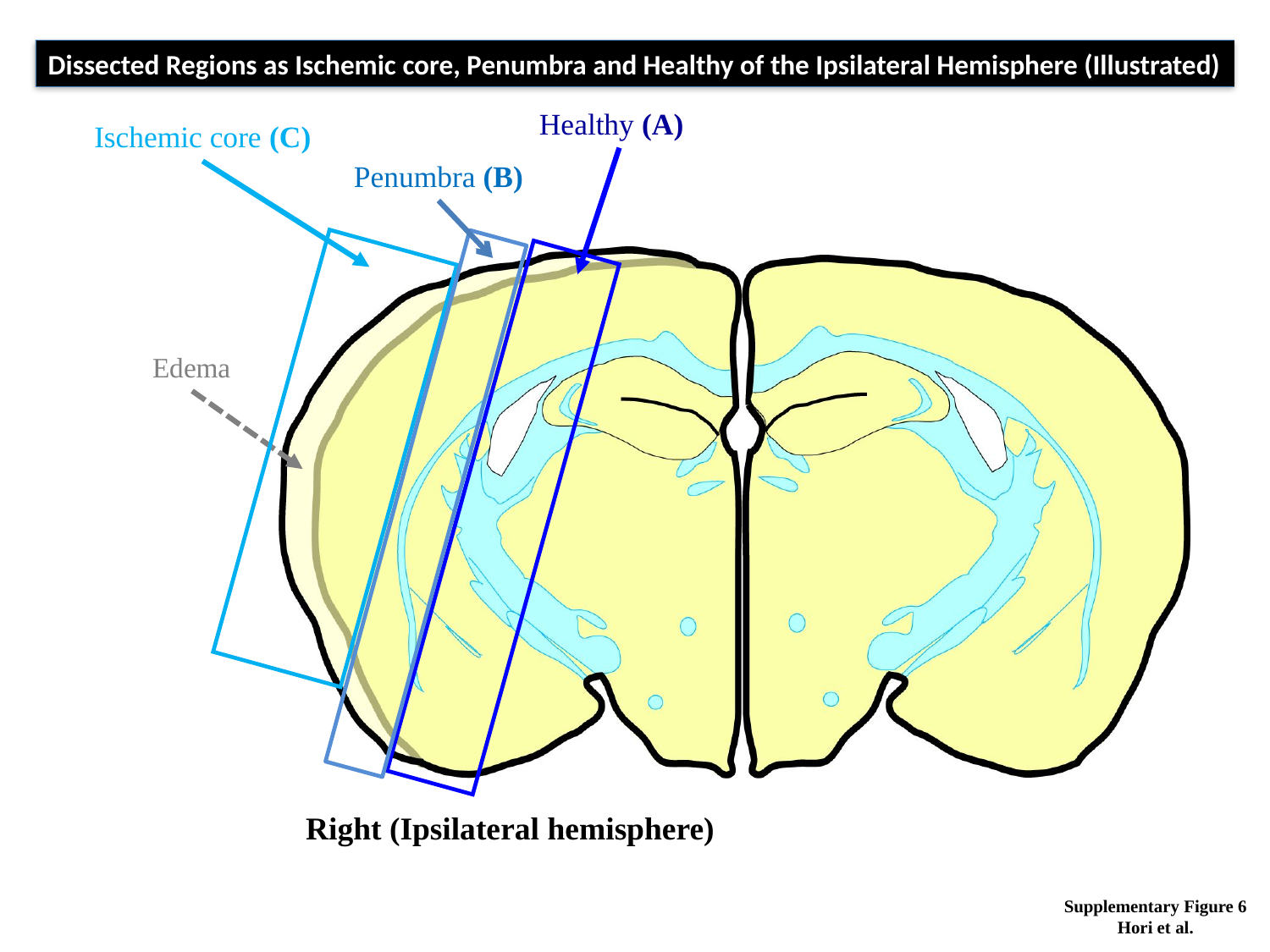

Dissected Regions as Ischemic core, Penumbra and Healthy of the Ipsilateral Hemisphere (Illustrated)
Healthy (A)
Ischemic core (C)
Penumbra (B)
Edema
Right (Ipsilateral hemisphere)
Supplementary Figure 6
Hori et al.
