# Supplementary material for: Transcriptomics and proteomics analyses of the PACAP38 influenced ischemic brain in permanent middle cerebral artery occlusion model mice
Source: J Neuroinflammation. 2012 Nov 23;9:256. doi: 10.1186/1742-2094-9-256 (PMC3526409; doi:10.1186/1742-2094-9-256)
Supplement: Additional file 17 — Figure S7. Western blot analysis of the anti-phosphorylated CRMP2 proteins cross-reacting proteins. [file 1742-2094-9-256-S17.pptx]

## Slide 1
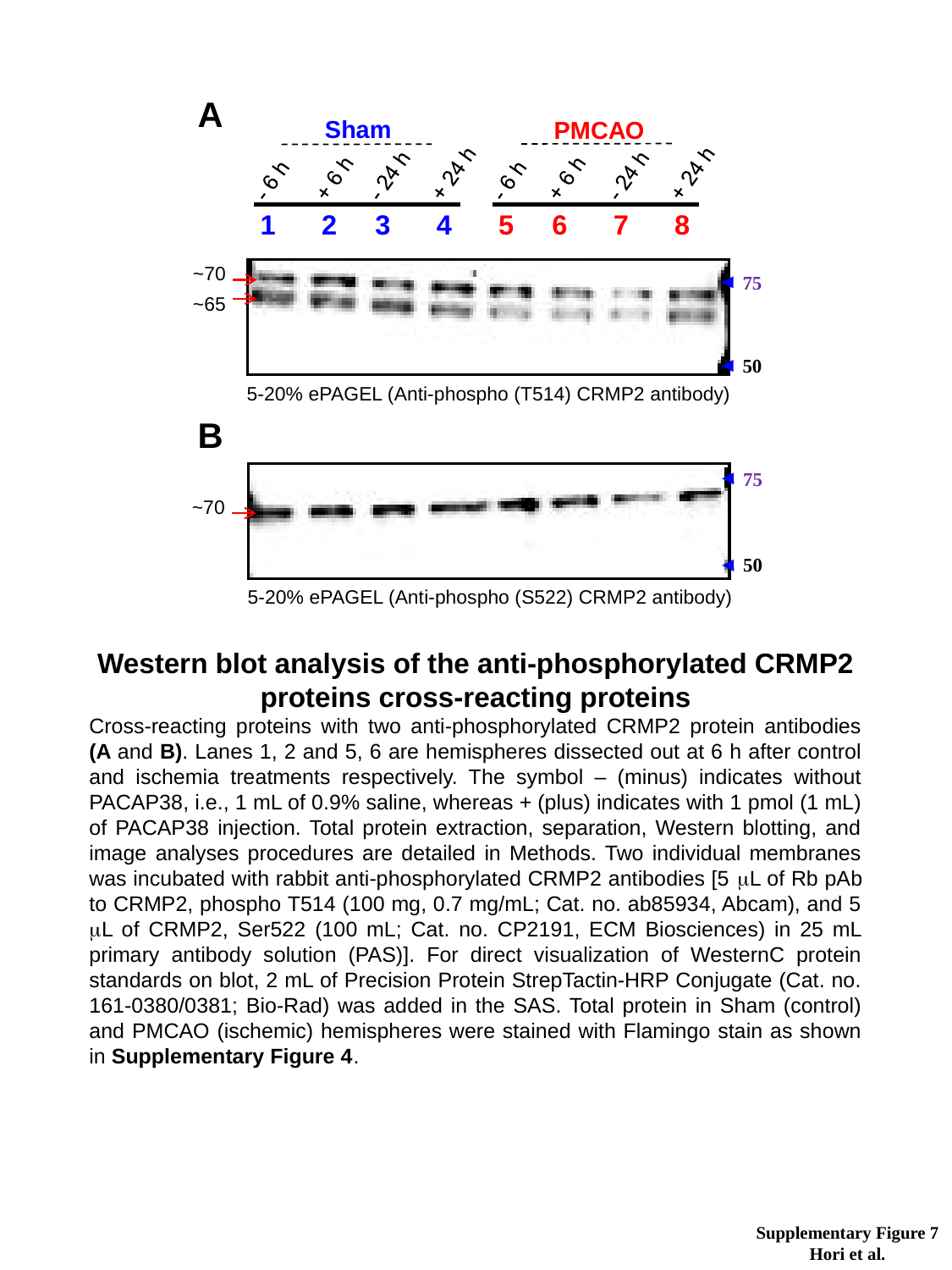

A
~70
75
~65
50
5-20% ePAGEL (Anti-phospho (T514) CRMP2 antibody)
B
75
~70
50
5-20% ePAGEL (Anti-phospho (S522) CRMP2 antibody)
Western blot analysis of the anti-phosphorylated CRMP2 proteins cross-reacting proteins
Cross-reacting proteins with two anti-phosphorylated CRMP2 protein antibodies (A and B). Lanes 1, 2 and 5, 6 are hemispheres dissected out at 6 h after control and ischemia treatments respectively. The symbol – (minus) indicates without PACAP38, i.e., 1 mL of 0.9% saline, whereas + (plus) indicates with 1 pmol (1 mL) of PACAP38 injection. Total protein extraction, separation, Western blotting, and image analyses procedures are detailed in Methods. Two individual membranes was incubated with rabbit anti-phosphorylated CRMP2 antibodies [5 mL of Rb pAb to CRMP2, phospho T514 (100 mg, 0.7 mg/mL; Cat. no. ab85934, Abcam), and 5 mL of CRMP2, Ser522 (100 mL; Cat. no. CP2191, ECM Biosciences) in 25 mL primary antibody solution (PAS)]. For direct visualization of WesternC protein standards on blot, 2 mL of Precision Protein StrepTactin-HRP Conjugate (Cat. no. 161-0380/0381; Bio-Rad) was added in the SAS. Total protein in Sham (control) and PMCAO (ischemic) hemispheres were stained with Flamingo stain as shown in Supplementary Figure 4.
Supplementary Figure 7
Hori et al.
